# Solid state organic amine detection in a photochromic porous metal organic framework†
†Electronic supplementary information (ESI) available: Experimental procedures and additional supporting data. CCDC 1024270 and 1024271. For ESI and crystallographic data in CIF or other electronic format see DOI: 10.1039/c4sc03224a



**DOI:** 10.1039/c4sc03224a

**Published:** 2014-12-05

**Authors:** Arijit Mallick, Bikash Garai, Matthew A. Addicoat, Petko St. Petkov, Thomas Heine, Rahul Banerjee

**Affiliations:** a Physical/Materials Chemistry Division , CSIR-National Chemical Laboratory , Dr Homi Bhabha Road , Pune 411008 , India . Email: r.banerjee@ncl.res.in ; Tel: +91 2025902535; b Academy of Scientific and Innovative Research (AcSIR) , New Delhi , India; c School of Engineering and Science , Jacobs University Bremen Campus Ring 1 , 28759 Bremen , Germany

## Abstract

A new Mg(ii) based photochromic porous metal–organic framework (MOF) has been synthesized bearing naphthalenediimide (NDI) chromophoric unit. This MOF (Mg–NDI) shows instant and reversible solvatochromic behavior in presence of solvents with different polarity. Mg–NDI also exhibits fast and reversible photochromism *via* radical formation. Due to the presence of electron deficient NDI moiety, this MOF exhibits selective organic amine (electron rich) sensing in solid state. The organic amine detection has been confirmed by photoluminescence quenching experiment and visual color change.

## Abstract 2

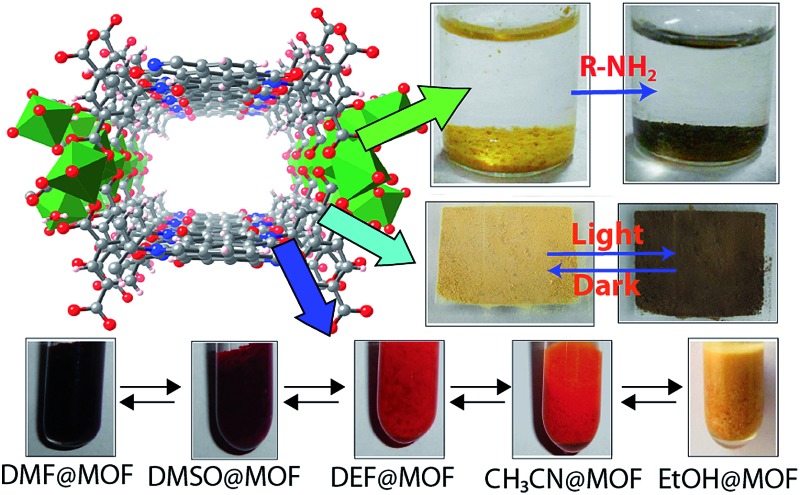
A new Mg(ii) based porous metal–organic framework (MOF) has been synthesized from naphthalenediimide (NDI) chromophoric unit containing linker. This MOF (Mg–NDI) shows instant and reversible photochromism as well as solvatochromic behavior. Due to the presence of electron deficient NDI moiety, this MOF exhibits selective organic amine (electron rich) sensing in solid state.

## Introduction

Transformation of the chemical information stored in a molecule into an analyzable signal has drawn researchers' attention for over two decades.^1^ A chemical sensor can detect an analyte through the change in color, luminescence, conductivity, *etc.* ^2^ However, analytes like explosives, hazardous chemicals, or gases, harmful radiations and bioactive reagents need to be detected in very low concentrations in order to avoid the danger inherent in them. In this regard, porous crystalline chemical sensors are quite interesting because of their quick, reversible and recyclable sensing ability.^3^ Metal organic frameworks (MOFs) are one important class of porous crystalline materials constructed by the co-ordination of metal ions with organic linkers.^4^ Such arrangement creates definite pores inside their structure allowing the incoming analyte to interact with the framework or another preloaded material. MOF based sensors generally interact with the incoming analytes in three different ways: (i) analyte–luminescent metal ion interaction,^5^ (ii) analyte–luminescent organic linker interaction,^6^ and (iii) analyte-trapped luminescent material interaction.^7^ Among these, the most efficient way to synthesize a MOF based chemical sensor is to utilize the analyte–organic linker interaction, because each analyte produces distinct signal upon interaction with the luminescent MOF linkers.

Herein, we report a porous solvatochromic MOF, constructed from the environmentally friendly element Mg(ii) and naphthalenediimide (NDI) based fluorescent linker, capable of sensing a diverse range of solvents in less than 60 seconds and having ability to detect the new incoming solvent during fast analyte–solvent exchange. Chemical entities such as organic amines have enormous importance in polymer, dye and pharmaceutical industries.^8^ However, most of these amines are colorless, making their differentiation *via* visual inspection difficult. These amines are also hazardous to the environment, and hence spillage of these materials should be sensed efficiently in order to prevent any probable harm. Traditional chemical sensors generally dissolve or decompose in presence of such analytes.^9^ Porous MOFs, on the other hand, bear an advantage over these traditional chemical sensors because of their heterogenous nature and ability to be used over multiple cycles. However, solvatochromic MOFs reported in the literature, have sluggish reversibility and require long detection time and/or sophisticated instrumental fabrication.^10^ Hence, it is still quite challenging to make a porous MOF which capable of sensing an analyte quickly and reliably. This Mg–NDI MOF, reported in this paper, is able to sense small sized amines by visual color change as well as photoluminescence quenching, making it a dual sensor of solvents and amines. Apart from these chemical entities, Mg–NDI is also able to detect the presence of radiations by showing reversible color change. This photochromism adds as another sensing property for Mg–NDI. To the best of our knowledge, this is the first report of a MOF where both solvatochromism and photochromism could be observed in one system. This type of dual sensing property is possible because of the presence of chromophoric unit forming the porous structure.

## Synthesis

This solvatochromic MOF (Mg–NDI) was synthesized from the solvothermal reaction of Mg(NO_3_)_2_·6H_2_O (24 mg, 0.093 mmol) and *N*,*N*′-bis(5-isophthalic acid)naphthalenediimide (H_4_BINDI, 21 mg, 0.035 mmol) in a mixture of DMF (4 mL) and 3 N HCl (0.2 mL) at 90 °C.

## Result and discussion

Mg–NDI MOF crystallized in *P*2/*c* space group and the secondary building unit (SBU) contains two different hexacoordinated Mg(ii) metal centers, two BINDI ligands, two coordinated DMF and one water molecule. The first Mg(ii) center is coordinated to the carboxylate oxygen atoms of isophthalate moieties present in BINDI ligand while the second Mg(ii) center is coordinated to three BINDI carboxylate oxygen atoms, two DMF and one water molecules. The PXRD pattern of the as-synthesized material matched well with the simulated pattern, except for the additional small peak at 2*θ* = 8.8°, possibly originated from the disordered solvent molecules present in the framework (Fig. S7 in ESI†). Mg–NDI retained its PXRD patterns after addition of analyte molecules (such as various solvents and amines), indicating the retention of crystallinity after incorporation of guest molecules in the framework. Generation of no additional peak in the PXRD pattern suggests that the solvent molecules are not coordinated to the metal ions, rather they are situated within the pores and weakly interacting with the pore walls to generate the necessary analyte response.

FT-IR analysis of the vacuum dried MOF (Fig. S9 in ESI†) shows sharp peaks at 1656 and 1550 cm^–1^ due to the presence of amide and carboxylate carbonyl functionalities, respectively. In comparison to the free ligand, this amide carbonyl stretching frequency (1708 cm^–1^) got shifted by 52 cm^–1^, possibly due to the change in dihedral angle from 128 to 103° in the crystal lattice (Fig. S5 in ESI†). Generation of additional broad peak at 3305 and sharp peaks at 2971 and 1044 cm^–1^ account for the O–H, C–H and C–O stretching frequencies for the EtOH present in EtOH@Mg–NDI. Such characteristic peaks (Fig. S9 in ESI†) were also observed for DMF, DEF, DMA incorporated MOFs (1649, 1642, 1607 cm^–1^, respectively for carbonyl stretching), DMSO@Mg–NDI (1015 cm^–1^ for S=O stretching) and MeCN@Mg–NDI (2253 cm^–1^ for C–N stretching). These weakly interacting solvent molecules do not affect the thermal stability of the MOF and are released from the framework between 80–150 °C during TGA which causes ∼15% weight loss of the material (Fig. S11 in ESI†). Mg–NDI framework is stable up to 300 °C under N_2_ atmosphere.

**Fig. 1:**
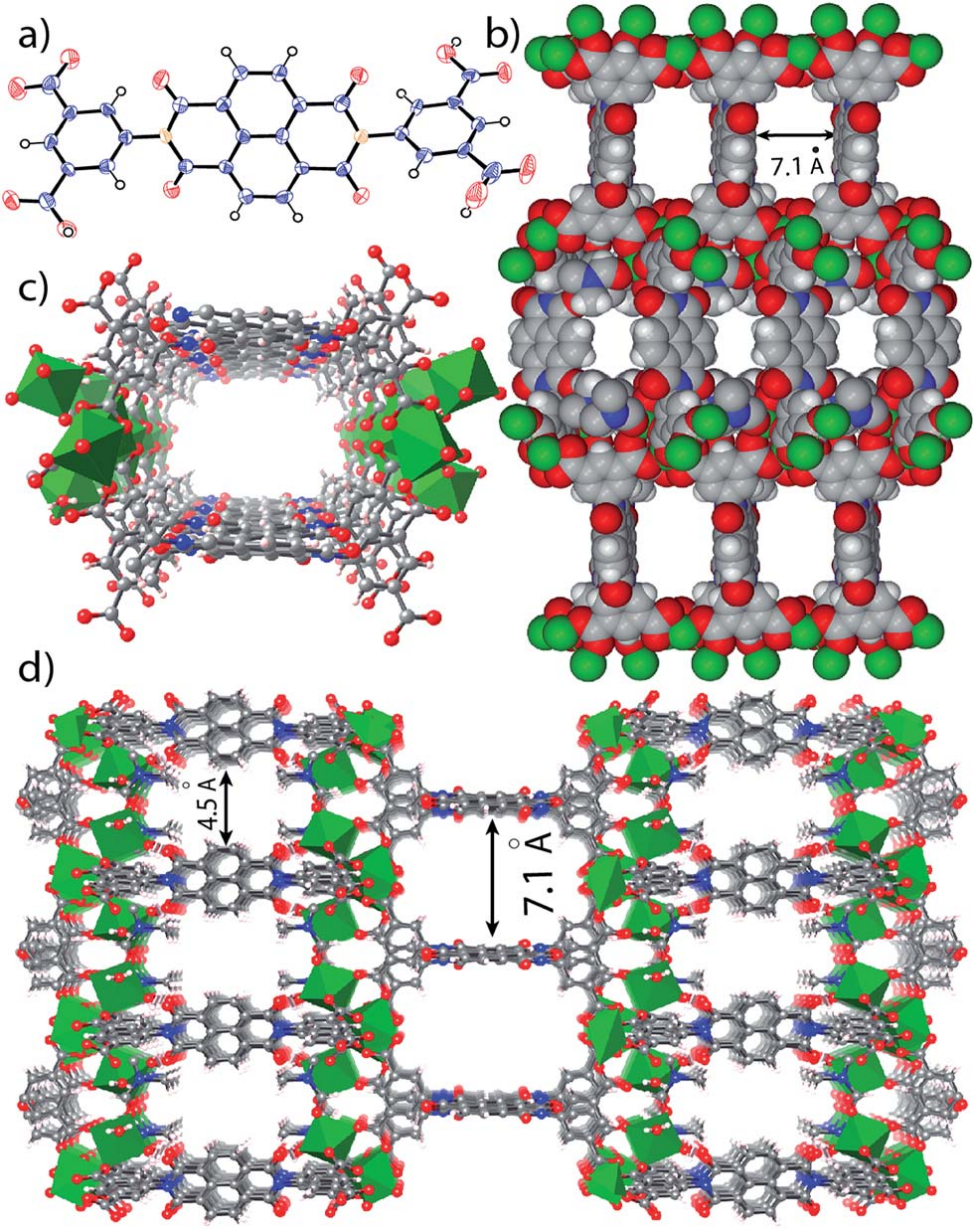
(a) ORTEP diagram of H_4_BINDI; (b) space filled diagram of Mg–NDI; (c) perspective view of a single pore and (d) perspective view of the extended structure of Mg–NDI.

**Fig. 2:**
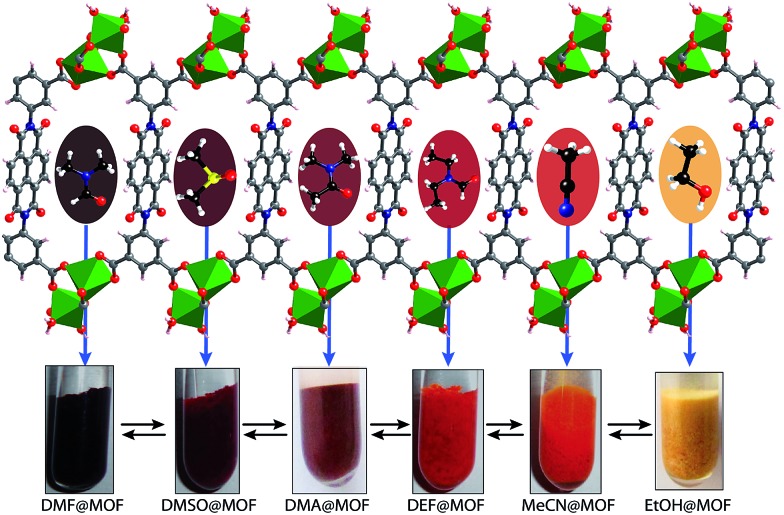
Schematic representation of Mg–NDI crystals showing different colors in different solvents. Hypsochromic shift was found to be observed with the increase in polarity of the solvent medium.

**Fig. 3:**
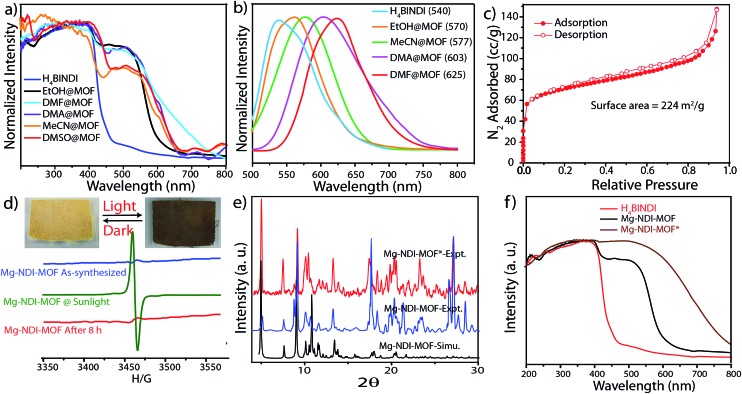
(a) Solid state UV-vis spectra, (b) solid state photoluminescence of Mg–NDI crystals soaked in different solvents; (c) N_2_ adsorption isotherm for Mg–NDI; (d) ESR spectra of Mg–NDI crystals before and after irradiation of sunlight; (inset) photograph representing the reversible photochromic behavior of Mg–NDI MOF crystal in sunlight and dark; (e) PXRD patterns of Mg–NDI crystals before and after irradiation of sunlight; (f) solid state UV-vis spectra of H_4_BINDI (red), Mg–NDI (black) and Mg–NDI* (brown).

In the crystal structure of free H_4_BINDI ligand, the chromophoric naphthalenediimide (NDI) moieties are π-stacked and separated by a distance of 3.3 Å (Fig. S6 in ESI†). However, within the MOF lattice, the moieties are separated by a longer distance of 7.1 Å (Fig. 1), preventing them from forming any π-stacked assembly. This spacing allows the NDI moieties to behave as discrete fragments, unlike H_4_BINDI ligand where the smaller spacing and existing π-interaction prohibit the analyte molecules from interacting with the chromophore. Analyte molecules can pass through the pores of Mg–NDI MOF, creating a definite interaction with the pore walls constructed by the BINDI linkers (Section S10 in ESI†). N_2_ adsorption analysis also indicated the presence of 224 m^2^ g^–1^ available surface area within this MOF (Fig. 3c). When the dark-brown colored as-synthesized MOF was placed in ethanol, the color of the resulting compound (EtOH@Mg–NDI) changed to bright-yellow. Similar solvent exchange with other solvents like DMSO, DEF, DMA, MeCN, generates different colors [brown, dark red, red, orange, respectively; Fig. 2] for the MOF depending on the polarity of the solvent. This color change is instant and reversible, *i.e.* after placing the MOF into the solvent; it converted into the corresponding distinct colored MOF in less than 60 seconds. This fact indicated that the interaction of the incorporated solvent and framework is short ranged and weak, allowing the external bulk solvents to easily replace the core solvent already present inside the porous framework.

Solid state UV-vis spectra revealed that the solvent incorporated Mg–NDI samples showed absorption in the visible range and absorption maxima are consistent with the solvent polarity. The UV-vis spectrum of both Mg–NDI and free H_4_BINDI ligand showed a strong absorption band at 370 nm (Fig. 3a), which corresponds to the n–π* and π–π* transition of the aromatic carboxylate ligands.^11^ In addition, a shoulder peak around 515 nm can also be observed in Mg–NDI, which could be attributed to an intermolecular electron-transfer transition. The UV-vis spectra of solvent@Mg–NDI (Fig. 3a) displayed a gradual broadening of absorption band in the region 515–680 nm with respect to different solvent polarity, which may arise from the intermolecular electron-transfer transition from solvent to BINDI linker within the MOF framework. The band gap energies of these solvent incorporated MOFs were calculated from UV-vis data which show that band gap energy of DMF@Mg–NDI and EtOH@Mg–NDI are 1.63 eV and 2.06 eV, respectively and are extreme values for all the tested solvents. The band gap energies of other solvent incorporated MOFs were found to increase with the increment of the solvent polarity (1.87, 1.88, 1.91, and 1.92 eV for DMA, DMSO, DEF, and MeCN, respectively; Fig. S12b in ESI†). Mg–NDI also exhibited solvent-dependent photoluminescence (PL). The PL spectrum of EtOH@Mg–NDI shows an emission at 570 nm and DMF@Mg–NDI shows an emission at 625 nm upon excitation at 515 nm, *i.e.* a 55 nm shift of *λ*_em_ was observed upon changing the solvent from EtOH to DMF. With increasing solvent polarity [DMF < DMSO < DEF < MeCN < EtOH] a gradual blue shift of *λ*_em_ was observed which correlates with the band gap energies of the solvent@Mg–NDI MOFs, showing that Mg–NDI MOF has a negative solvatochromic effect. The solid H_4_BINDI ligand showed the photoluminescence emission maxima at 540 nm upon excitation at 370 nm (Fig. 3b). Whereas, the PL spectra of the dry Mg–NDI MOF showed strong emission bands centered at *λ*_em_ = 570 nm upon excitation at 515 nm. The probable reason behind the strong red shift of emission maxima is the coupling interaction between the neighboring ligands through Mg(ii) inside the framework. Thus, the solvatochromism of Mg–NDI MOF is much better than that of the H_4_BINDI ligand itself.

It is noteworthy that Mg–NDI also shows sensitivity towards sunlight irradiation. The yellow colored crystals of Mg–NDI undergo photochromic transition and become black within five minutes. The black crystals then regain their yellow color when kept in dark for 8 hours at ambient condition. PXRD data of Mg–NDI collected before and after the photochromic transition are exactly identical (Fig. 3e) which proves that no structural change is caused because of the irradiation. However, the UV-vis spectra show an abrupt change in absorption band (Fig. 3f) which reveals that reversible photochromism may be resulted from an electron-transfer chemical process inside the structure, and not from a structural transformation. The UV-vis spectrum of the irradiated Mg–NDI (Mg–NDI*) displays a transformation of the tiny shoulder peak at 515 nm for Mg–NDI, into a broad band in the region 515–700 nm (Fig. 3f). This broadening is arising from the photo-induced electron-transfer transition in Mg–NDI*. NDI moiety is known to be redox-active and can generate radicals upon light irradiation.^11^ Therefore, besides π–π electron-transfer, this photochromic process may also arise from the photo-induced radical generation of organic ligand. This radical generation has been confirmed by ESR spectra. Mg–NDI exhibits no ESR signal, but Mg–NDI* shows a single-peak radical signal with a *g* value of 1.9514 (Fig. 3d).

**Fig. 4:**
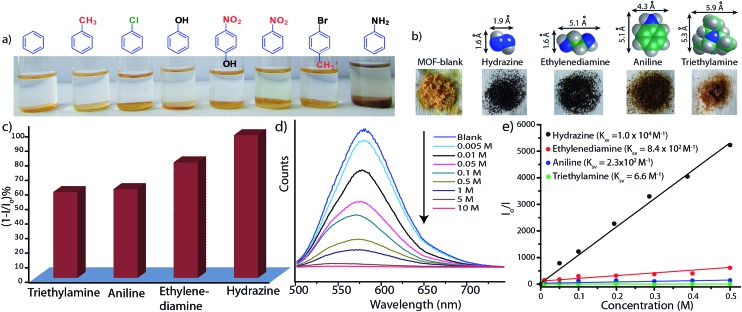
(a) Photograph of selective detection of aniline over other aromatic functional molecules; (b) photograph showing the color change of Mg–NDI samples in presence of different amines (0.1 M); (c) bar chart representation for quenching efficiency of Mg–NDI in presence of different amines (0.01 M) in EtOH; (d) reduction of PL emission intensities of Mg–NDI by gradual increase in concentration of aniline; (e) Stern–Volmer plot for Mg–NDI with different amines.

As the Mg–NDI framework is electron deficient in nature due to the presence of NDI chromophore, we decided to check its ability to sense electron rich chemical species. Among several electron rich chemical species, this MOF can selectively sense small sized organic amines by visual color change as well as quenching of its fluorescence efficiency. Electron rich organic amines can form charge transfer complex with the NDI moieties within the framework, resulting a change in color as well as photoluminescence property. Treatment of Mg–NDI with various organic amines like aniline, hydrazine, ethylene diamine, triethylamine, dimethylamine, 1,3-propanediamine, ethylamine, methylamine showed a distinct color change (to black) over other functionalized analytes like chlorobenzene, toluene, benzene, phenol, 4-nitrophenol, nitrobenzene, 4-bromotoluene, *etc.* (Fig. 4a). This color change is extremely rapid and very prominent and can be easily detected from naked eye inspection. Mg–NDI is able to detect the presence of amine from a very low concentration (10^–5^ M) in the solid state.

**Fig. 5:**
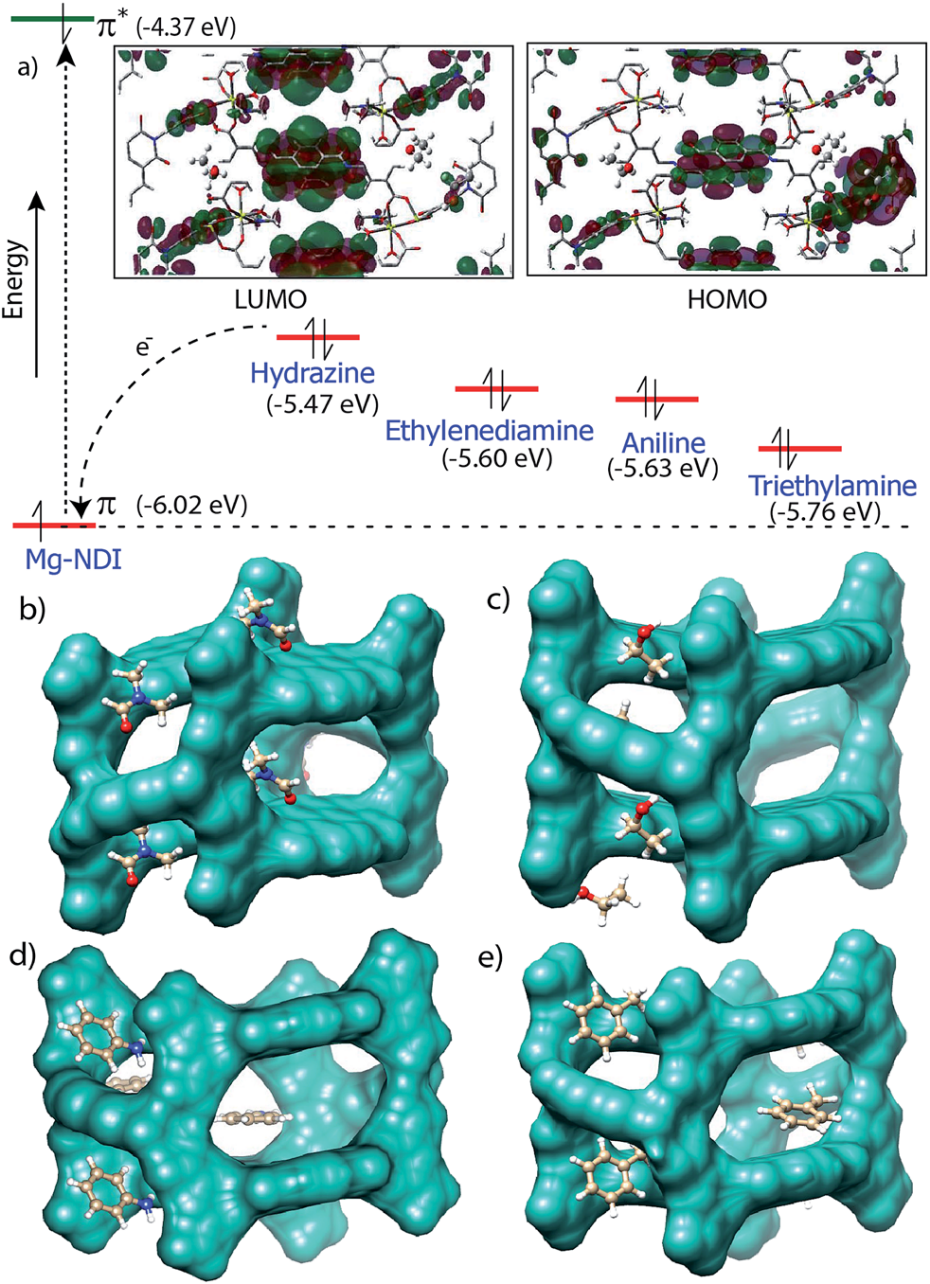
(a) HOMO (π) and LUMO (π*) energy levels of Mg–NDI and different amines. The inset shows contour plots of the HOMO and LUMO for Mg–NDI. Energy optimized structures of (b) DMF, (c) EtOH, (d) aniline and (e) toluene incorporated Mg–NDI has been represented.

Chemical sensing using as-synthesized solid MOF samples is rarely reported in the literature. In all the previous reports, analyte sensing has been done with a suspension of the sensor MOF, or by making a thin film probably due to their poor efficiency.^12^ Presence of the chromophoric NDI moiety has made Mg–NDI capable to sense organic amines in solid state. To explore the ability of Mg–NDI to sense a trace quantity of amine, fluorescence-quenching experiment were performed with addition of analytes, with increasing concentration to the fixed amount Mg–NDI. Rapid and strong fluorescence quenching was observed upon increasing the concentration of amine solution. The MOF was tested for its sensing ability against a wide range of amines like aniline, ethylenediamine, triethylamine, hydrazine, *etc.* where an instant color change of the solid material was observed for the case of hydrazine, methylamine, ethylamine, dimethylamine and ethylenediamine. In the cases of aniline and triethylamine, the rate of color change was found to be relatively slow because of their bulky size. The PL spectra revealed that the tested amines showed prominent PL quenching response over other analytes which can also be detected by visual color change to black. The fluorescence quenching can be attributed to the donor–acceptor electron transfer between amines and MOF. To understand the electron transfer process, the HOMO and LUMO energy of Mg–NDI was determined by cyclic voltammetry measurement (Fig. S23 in ESI†). The electron transfer happens because the HOMO energy (–6.02 eV) of Mg–NDI is lower than that of amine analytes [hydrazine: –5.47 eV, ethylenediamine: –5.60 eV, aniline: –5.63 eV and triethylamine: –5.76 eV]. The electron transfer from hydrazine to Mg–NDI is faster than other amines because of higher energy difference as compare to other amines (Fig. 5a). In case of phenol the PL quenching is also observed due to the presence of electron rich phenolic hydroxy groups, but no color change has been observed in visible light. Few experiments have been designed to check vapor phase sensing ability of Mg–NDI. In a typical experiment, the vacuum dried Mg–NDI was exposed to the vapor of the amines (obtained from the evaporation of the pure liquid amines) for a period of ∼30 min and then measured their photoluminescence property. It was observed that because of the exposure to the amine vapor, photoluminescence property of Mg–NDI has been completely quenched, as that for amines from solution (Section S9 in ESI†). Thus, Mg–NDI is also able to show vapor phase sensing for amines.

In order to get better understanding of the solvatochromic behavior and the corresponding PL quenching of Mg–NDI, DFT calculations were performed for bare and analyte incorporated MOFs, (Section S12 in ESI†). Optimized structures of the solvent incorporated MOFs (Fig. 5b and c) suggest that the solvent molecules have a weak dipolar interaction with the pore walls. Band gap energy for DMF@Mg–NDI and EtOH@Mg–NDI were found to be 1.32 and 1.41 eV, respectively. The calculated band gap values, calculated while keeping all framework atoms fixed in their crystallographic positions, are in accordance with the corresponding experimental band-gap values.

## Conclusion

In conclusion, a new metal organic framework (Mg–NDI) has been synthesized using a NDI-based chromophoric linker. Presence of the NDI moiety inside the framework made Mg–NDI capable of showing photochromic as well as solvatochromic property. Mg–NDI is able to demonstrate a quick (within 60 seconds) and reversible solvatochromic behavior in presence of solvents having different polarities. The photoluminescence emission maximum shows hypsochromic shift according to the increase in solvent polarity. Interestingly, the band gap of the solvent incorporated material was found to show a linear relationship with the solvent polarity. Mg–NDI is also exhibit photochromism *via* electron transfer pathway because of the presence of the redox active organic linker. Mg–NDI also exhibits colorimetric and fluorescence sensor of small sized amine molecules. Mg–NDI is able to selectively sense amine molecules, from a series of similar sized analytes, through prominent color change. Sensing of the mixed solvents detection is underway in our laboratory.
