# Supplementary material for: Solid state organic amine detection in a photochromic porous metal organic framework
Source: Chem Sci. 2014 Dec 5;6(2):1420–5. doi: 10.1039/c4sc03224a (PMC5811159; doi:10.1039/c4sc03224a)
Supplement: Supplementary file 1 [file SC-006-C4SC03224A-s001.pdf]

Electronic Supplementary Material (ESI) for Chemical Science.
This journal is © The Royal Society of Chemistry 2014

Electronic Supporting Information

Solid State Organic Amine Detection in a Photochromic Porous Metal Organic Framework

Arijit Mallick,[a] Bikash Garai,[a] Matthew A. Addicoat,[b] Petko St. Petkov,[b] Thomas Heine[b] and Rahul Banerjee[a]*

[a]Physical/Materials Chemistry Division, CSIR- National Chemical Laboratory, Dr. Homi Bhabha Road, Pune 411008, India.

[b]School of Engineering and Science, Jacobs University Bremen Campus Ring 1, 28759 Bremen, Germany.

Email: r.banerjee@ncl.res.in; Fax: + 91-20-25902636, Tel: + 91-20-25902535

Table of Contents

Section	Subject	Page
S1	Materials and methods	3
S2	Synthesis of ligand and metal organic framework	4
S3	Single crystal XRD and crystal structures of **Mg-NDI** and **H_4BINDI**	6
S4	PXRD patterns of the materials	14
S5	FT-IR spectroscopy of materials	16
S6	TG analyses of the materials	18
S7	UV-vis spectroscopic measurements	19
S8	Confocal microscopy imaging	20
S9	Photoluminescence (PL) analysis	21
S10	N_2 adsorption measurements	27
S11	Electrochemical measurements	29
S12	DFT calculation for Mg-NDI	30
S13	References	31

S1: Materials and methods

All reagents were commercially available and used as received. Powder X-ray diffraction (PXRD) patterns were recorded on a Rigaku Smartlab diffractometer for Cu K_α radiation (λ = 1.5406 Å), with a scan speed of 2° min^{-1} and a step size of 0.02° in 2θ. Fourier transform infrared (FT-IR) spectra were taken on a Bruker Optics ALPHA-E spectrometer with a universal Zn-Se ATR (attenuated total reflection) accessory in the 600-4000 cm^{-1} region or using a Diamond ATR (Golden Gate). Thermo-gravimetric' analyses (TGA) were carried out on a SDT Q600 TG-DTA analyzer under N_2 atmosphere at a heating rate of 5 ºC min^{-1} within a temperature range of 40-900 °C. All gas adsorption experiments (up to 1 bar) were performed on a *Quantachrome Autosorb* automatic volumetric instrument. UV-vis absorbance studies were carried out with Varian Carry 50 instrument equipped with a single beam facility (with a spectral resolution of 0.5 nm). Horiba Jobin Yvon Fluorolog 3 spectrophotometer having 450 W Xenon lamp was used for steady state fluorescence studies.

S2: Synthesis of ligand and metal organic framework

N,N'-bis(5-isophthalic acid)naphthalenediimide (H_4BINDI) was synthesized according to a previously reported procedure[1]. 1,4,5,8-tetracarboxydianhydride (1.34 g, 5.0 mmol) was taken into a 250 mL round bottomed flask and suspended in 25 mL acetic acid. The mixture was stirred for 10 min. To this solution, 5-aminoisophthalic acid (1.81 g, 10.0 mmol) was added and the solution allowed reflux for 12 h. The reaction was allowed to cool to room temperature and water (20 ml) was added to precipitate the product. The product was collected by filtration washed with ethanol and dried in vacuum to yield 2.4 g of off-white solid (isolated yield = 2.4 g, 77%). The compound was recrystallized from DMF as an off-yellow materials (isolated yield = 2.1 g, 67%).

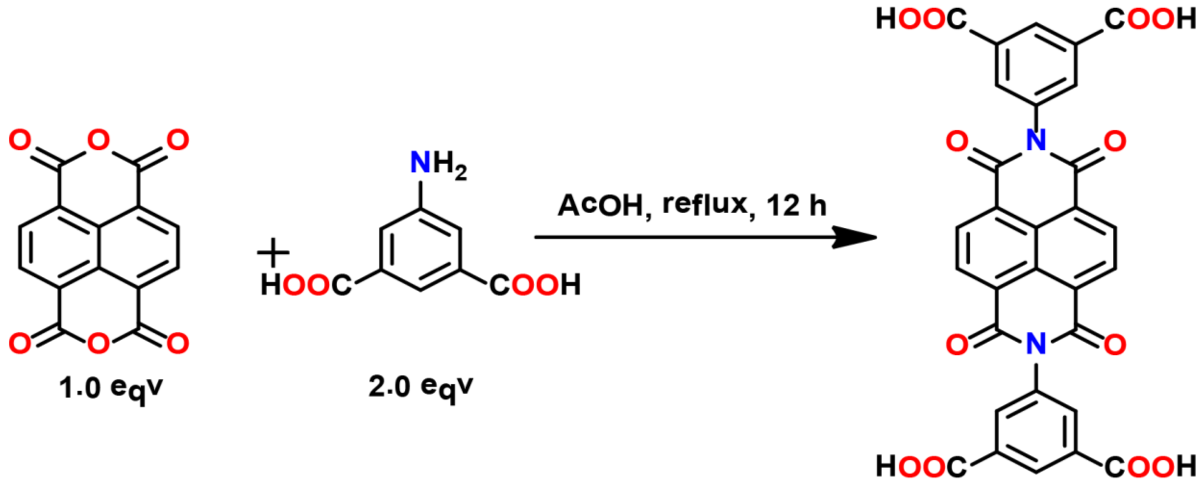

Figure S1: Scheme for synthesis of H_4BINDI.

Synthesis of Mg-NDI: The plate-like crystals of Mg-NDI MOF was synthesized by reacting *N,N'*-bis(5-isophthalic acid)naphthalenediimide (H_4BINDI) (21 mg, 0.035 mmol) with $Mg(NO_3)_2.6H_2O$ (24 mg, 0.093 mmol) in 4 mL DMF and 0.2 mL HCl (3 N) at 90 ˚C for 24 h. Orange color crystals were separated after the reaction and preserved in dry DMF/DEF for further applications/characterizations.

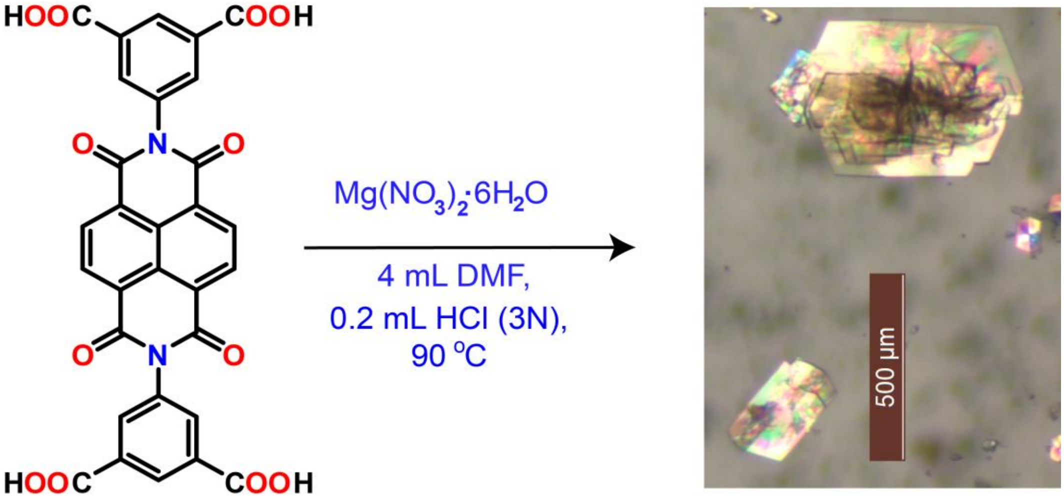

Figure S2: Schematic representation for synthesis of Mg-NDI.

S3: Single crystal XRD and crystal structures of Mg-NDI and H_4BINDI

As synthesized crystals of **Mg-NDI** and **H_4BINDI** were coated with paratone-N and placed on top of a nylon cryoloop (Hampton research) and then mounted in the diffractometer. The data collection was done at 150 and 293 K, respectively. The crystals were mounted on a Super Nova Dual source X-ray Diffractometer system (Agilent Technologies) equipped with a CCD area detector and operated at 250 W power (50 kV, 0.8 mA) to generate Mo Kα radiation (λ = 0.71073 Å) and Cu Kα radiation (λ = 1.54178 Å) at 298(2) K. Initial scans of each specimen were performed to obtain preliminary unit cell parameters and to assess the mosaicity (breadth of spots between frames) of the crystal to select the required frame width for data collection. $CrysAlis^{Pro}$ program software was used suite to carry out overlapping φ and ω scans at detector (2θ) settings (2θ = 28). Following data collection, reflections were sampled from all regions of the Ewald sphere to redetermine unit cell parameters for data integration. In no data collection was evidence for crystal decay encountered. Following exhaustive review of collected frames the resolution of the data set was judged. Data were integrated using $CrysAlis^{Pro}$ software with a narrow frame algorithm. Data were subsequently corrected for absorption by the program SCALE3 ABSPACK[2] scaling algorithm.

These structures were solved by direct method and refined using the SHELXTL 97[3] software suite. Atoms were located from iterative examination of difference F-maps following least squares refinements of the earlier models. Final model was refined anisotropically (if the number of data permitted) until full convergence was achieved. Hydrogen atoms were placed in calculated positions (C-H = 0.93 Å) and included as riding atoms with isotropic displacement parameters 1.2-1.5 times Ueq of the attached C atoms. In some cases modeling of electron density within the voids of the frameworks did not lead to identification of recognizable solvent molecules in these structures, probably due to the highly disordered contents of the

large pores in the frameworks. Highly porous crystals that contain solvent-filled pores often yield raw data where observed strong (high intensity) scattering becomes limited to ~1.0 Å at best, with higher resolution data present at low intensity. A common strategy for improving X-ray data, increasing the exposure time of the crystal to X-rays, did not improve the quality of the high angle data in this case, as the intensity from low angle data saturated the detector and minimal improvement in the high angle data was achieved. Additionally, diffused scattering from the highly disordered solvent within the void spaces of the framework and from the capillary to mount the crystal contributes to the background and the 'washing out' of the weaker data. Electron density within void spaces has not been assigned to any guest entity but has been modeled as isolated oxygen and/or carbon atoms. The foremost errors in all the models are thought to lie in the assignment of guest electron density. The structure was examined using the *ADSYM* subroutine of PLATON[4] to assure that no additional symmetry could be applied to the models. The ellipsoids in ORTEP diagrams are displayed at the 50% probability level unless noted otherwise.

Generally MOF structures contain large amount voids in their extended structure, and this porous nature of the framework is enhanced in case of the 3D MOFs. This causes the origin of errors indicating the presence of large solvent accessible voids inside the MOF. This types of errors is common for the case of porous materials structure and thus the presence of error PLAT602_ALERT_2_A (VERY LARGE Solvent Accessible VOID(S) in Structure) is justified for Mg-NDI structure. Add to this, Mg-MDI is composed of lightweight Mg-atoms (Mg is having density1.73 g/cc) apart from the lightweight of C-atoms and each unit cell (Volume: 6082.58 $Å^3$) having 8 Mg-centres present. Thus, because of the presence of large void space and the lightweight elements, density of the material is less than 1.0 gcm^{-3} and this shows up as a B level error 'PLAT049_ALERT_1_B (Calculated Density less than 1.0 gcm^{-3})' in the

checkcif report for Mg-NDI. And, because of the highly porous structure of Mg-NDI, despite of our numerous efforts to assign the residual electron density and some distortion retained in the crystal structure. Thus, this less accurate modelling of the MOF structure generates other B level errors like PLAT220_ALERT_2_B , PLAT222_ALERT_3_B, PLAT230_ALERT_2_B, PLAT234_ALERT_4_B, PLAT242_ALERT_2_B and PLAT420_ALERT_2_B.

CCDC 1024270 and CCDC 1024271 contain the crystallographic data for **H_4BINDI** and **Mg-NDI** respectively.

Table S1: Crystal data and structure refinement for H_4BINDI

Identification code	H4BINDI
Empirical formula	$C_{279.28}H_{164}N_{37}O_{100}$
Formula weight	5637.84
Temperature/K	293(2)
Crystal system	monoclinic
Space group	*C*2/*c*
a/Å	33.067(4)
b/Å	14.8252(15)
c/Å	17.1809(12)
α/°	90.00
β/°	121.19(10)
γ/°	90.00
Volume/Å^3	7205.1(12)
Z	1
ρ_{calc}mg/mm^3	1.401
m/mm^{-1}	0.101
F(000)	2898.7
Crystal size/mm^3	0.41 × 0.40 × 0.10
Theta range for data collection	6.16 to 58.4°
Index ranges	$-43 \leq h \leq 37$, $-11 \leq k \leq 20$, $-23 \leq l \leq 13$
Reflections collected	11966
Independent reflections	9756[R(int) = 0.0669]
Data/restraints/parameters	9756/0/499
Goodness-of-fit on F^2	1.113
Final R indexes [I>2σ (I)]	R_1 = 0.1429, wR_2 = 0.2897
Final R indexes [all data]	R_1 = 0.1780, wR_2 = 0.2988
Largest diff. peak/hole / e Å^{-3}	1.024/-0.509

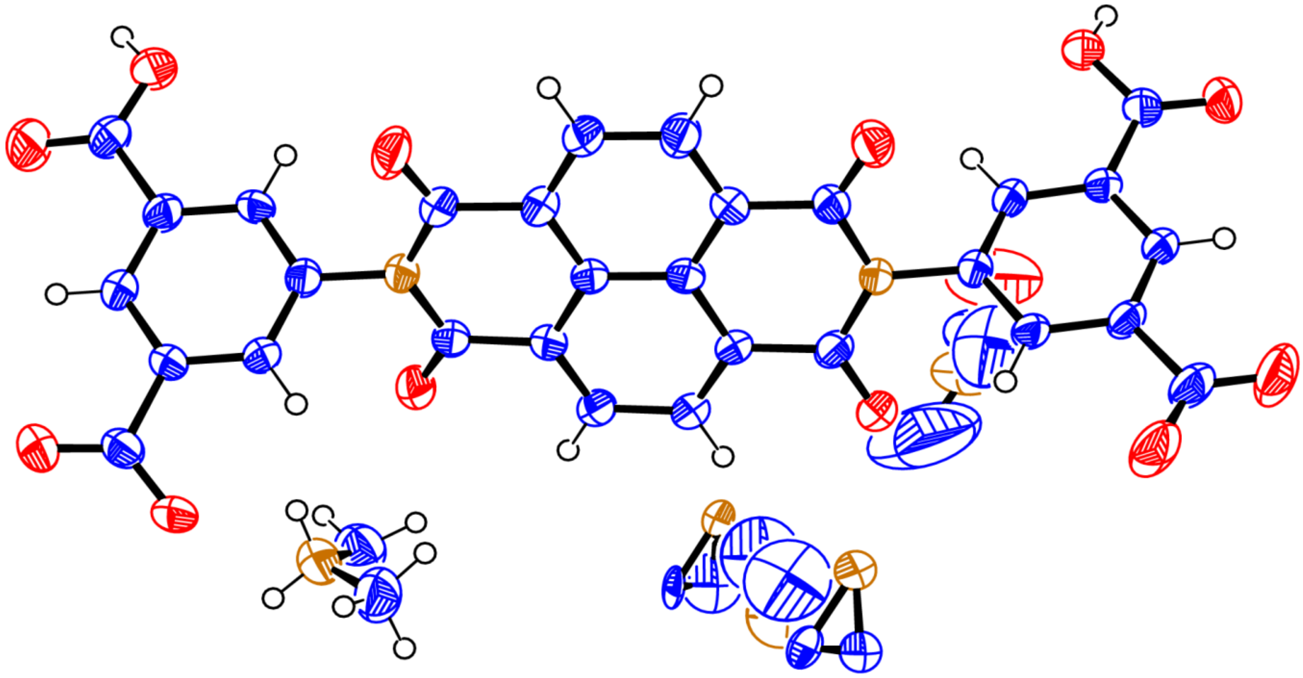

Figure S3: ORTEP drawing of H_4BINDI. Thermal ellipsoids set to 50% probability level.

Table S2: Crystal data and structure refinement for Mg-NDI

Identification code	Mg-NDI
Empirical formula	$C_{69}H_{35}Mg_4N_7O_{30}$
Formula weight	1539.28
Temperature/K	150(2)
Crystal system	monoclinic
Space group	*P*2/*c*
a/Å	34.3413(18)
b/Å	10.0447(7)
c/Å	17.7382(17)
α/°	90
β/°	96.234(6)
γ/°	90
Volume/Å^3	6082.6(8)
Z	2
ρ_{calc} mg/mm^3	0.8404
m/mm^{-1}	0.085
F(000)	1572.0
Crystal size/mm^3	0.40 × 0.20 × 0.20
Theta range for data collection	5.96 to 58.38°
Index ranges	$-43 \leq h \leq 47$, $-12 \leq k \leq 13$, $-23 \leq l \leq 22$
Reflections collected	34619
Independent reflections	14116[R(int) = 0.0896]
Data/restraints/parameters	14116/0/506
Goodness-of-fit on F^2	0.7929
Final R indexes [I>2σ (I)]	$R_1 = 0.1057$, $wR_2 = 0.2679$
Final R indexes [all data]	$R_1 = 0.1637$, $wR_2 = 0.2975$
Largest diff. peak/hole / e Å^{-3}	0.7875/-0.5894

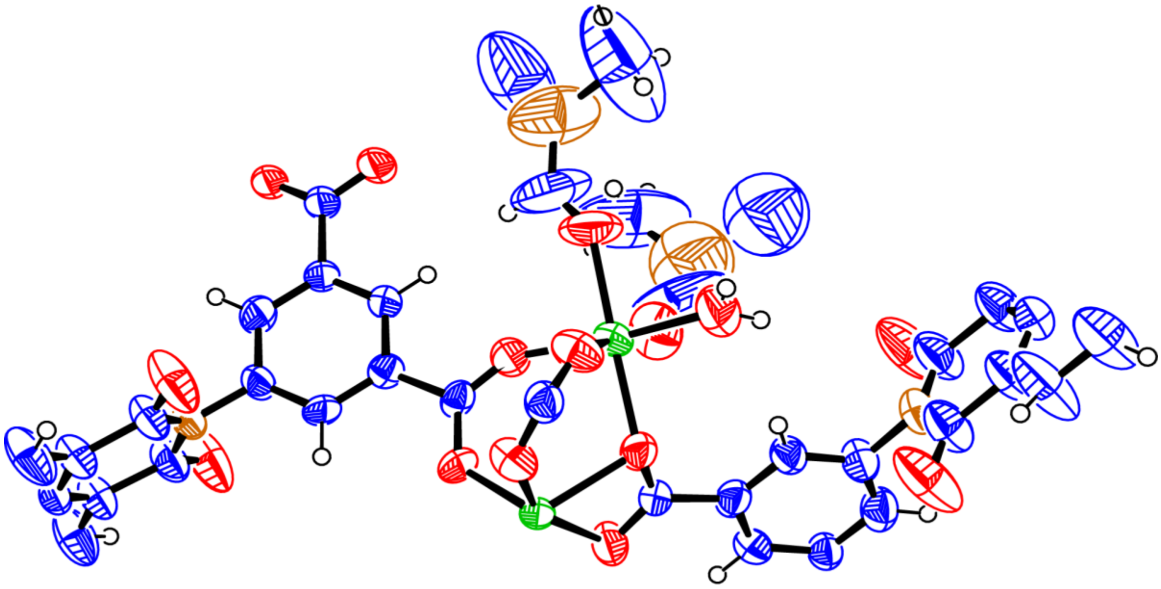

Figure S4: ORTEP drawing of **Mg-NDI**. Thermal ellipsoids set to 50% probability level.

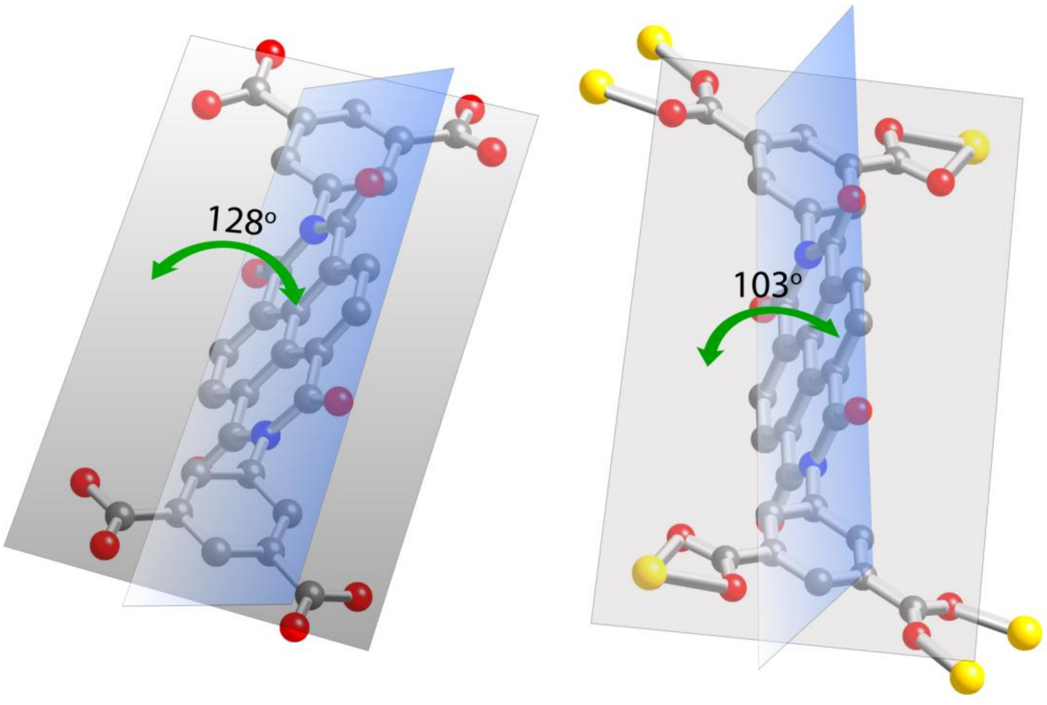

Figure S5: The dihedral angle between naphthalene ring and isophthalic acid moiety in (a) H_4BINDI and (b) Mg-NDI framework.

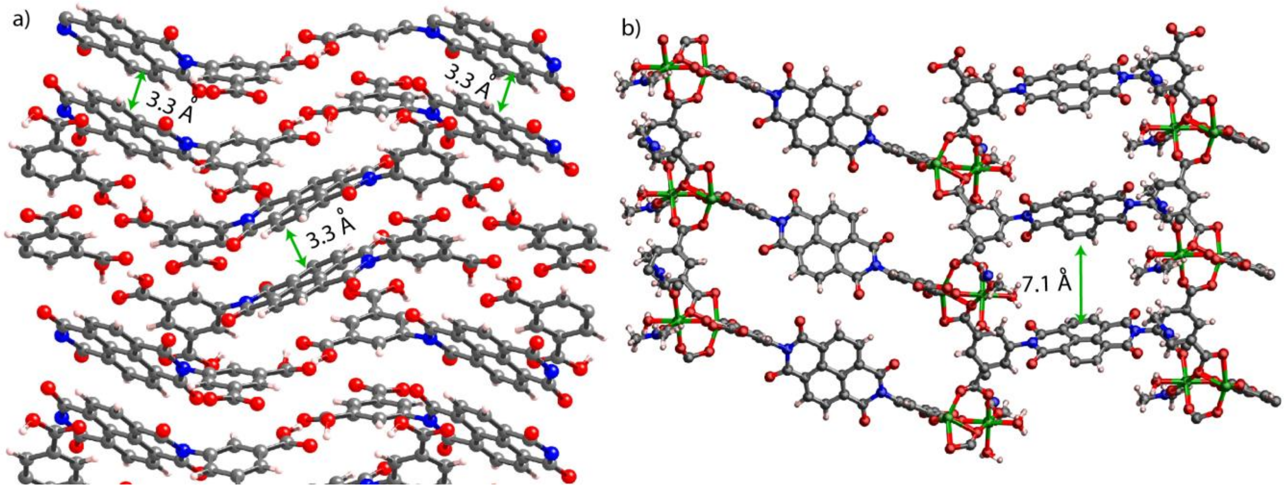

Figure S6: Single crystal structure and packing diagram of a) H_4BINDI showing the π–π interaction between NDI moieties (3.3 Å) and b) Mg-NDI showing the absence of π–π interaction between NDI moieties (7.1 Å).

S4: PXRD patterns of the materials

As synthesized crystals of **Mg-NDI** were taken out from the mother solution and washed with dry DMF and pure ethanol and then dried in vacuum. The dried materials were soaked in different solvents and PXRD measurement was performed to check the stability of the MOFs.

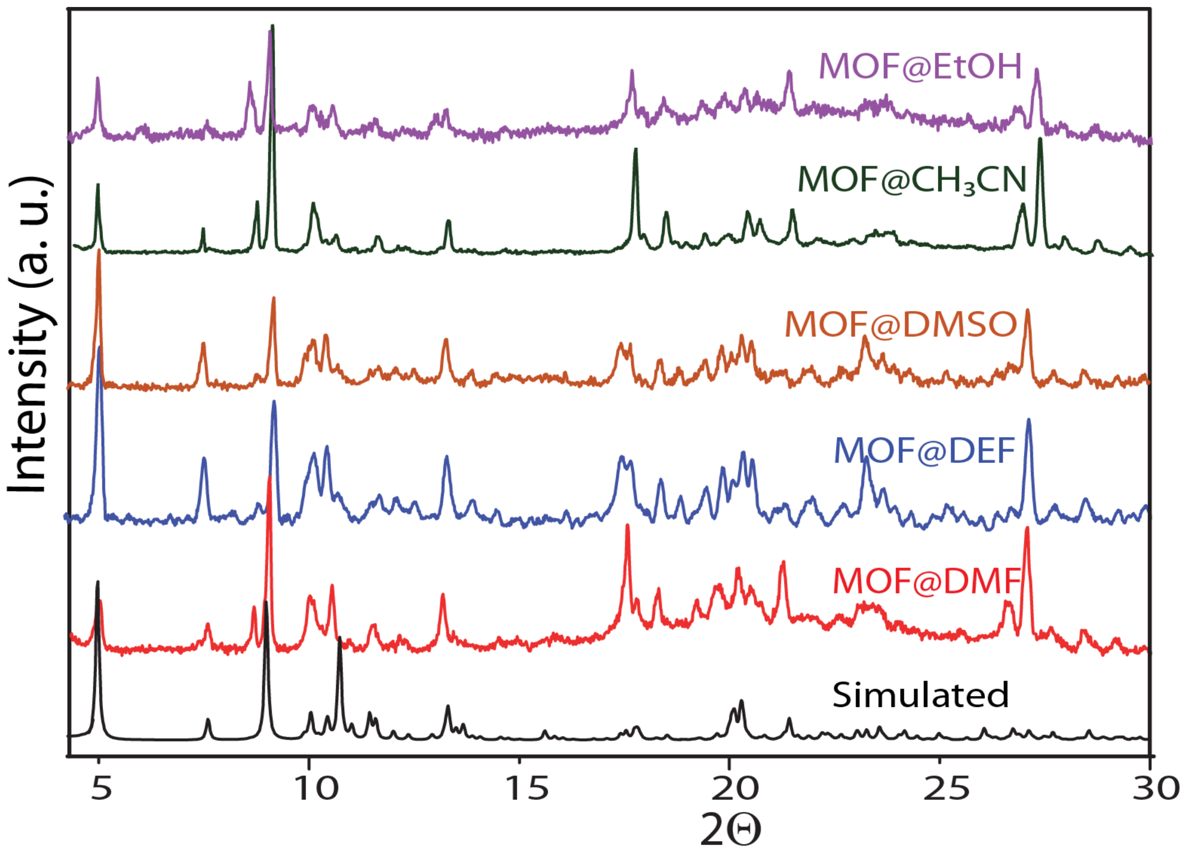

Figure S7: PXRD pattern of solvent@Mg-NDI compared with the simulated pattern of Mg-NDI.

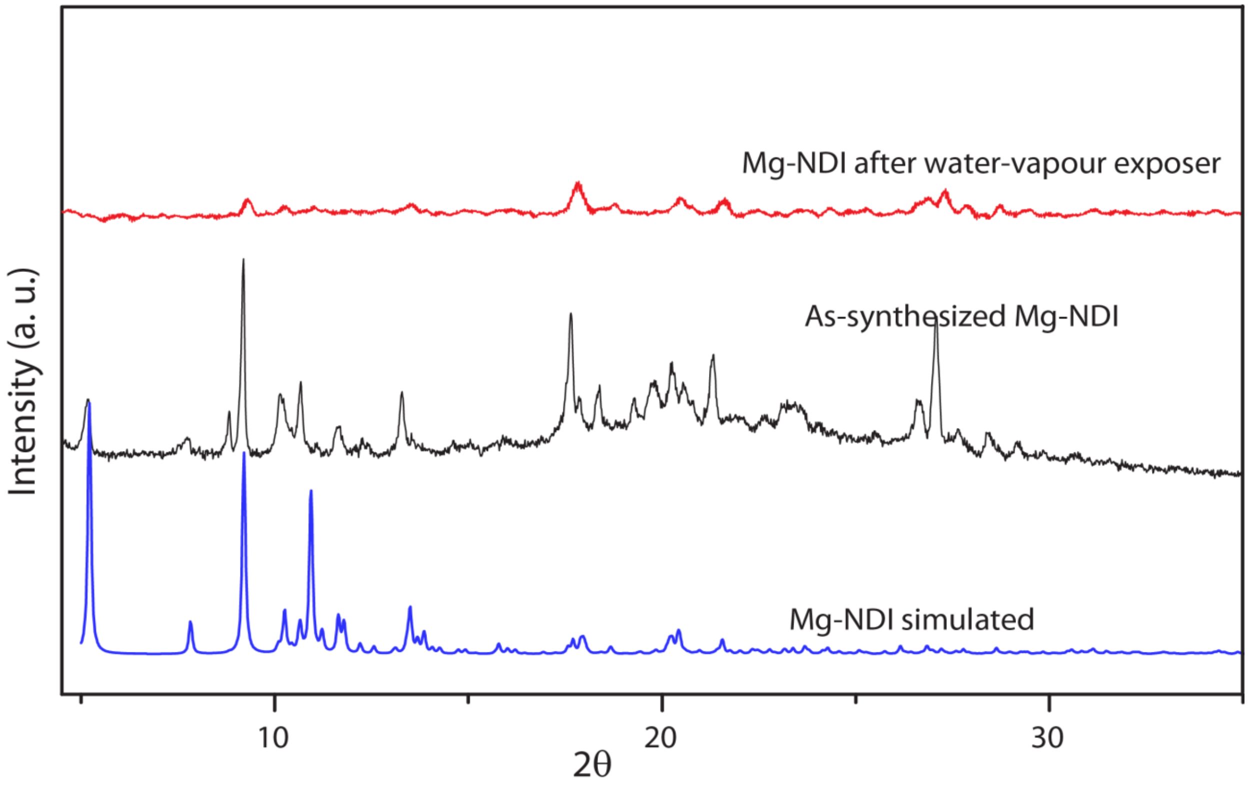

Figure S8: PXRD pattern of water vapor treated Mg-NDI compared with the simulated and as-synthesized pattern of Mg-NDI.

S5: FT-IR spectroscopy of H_4BINDI and metal organic framework

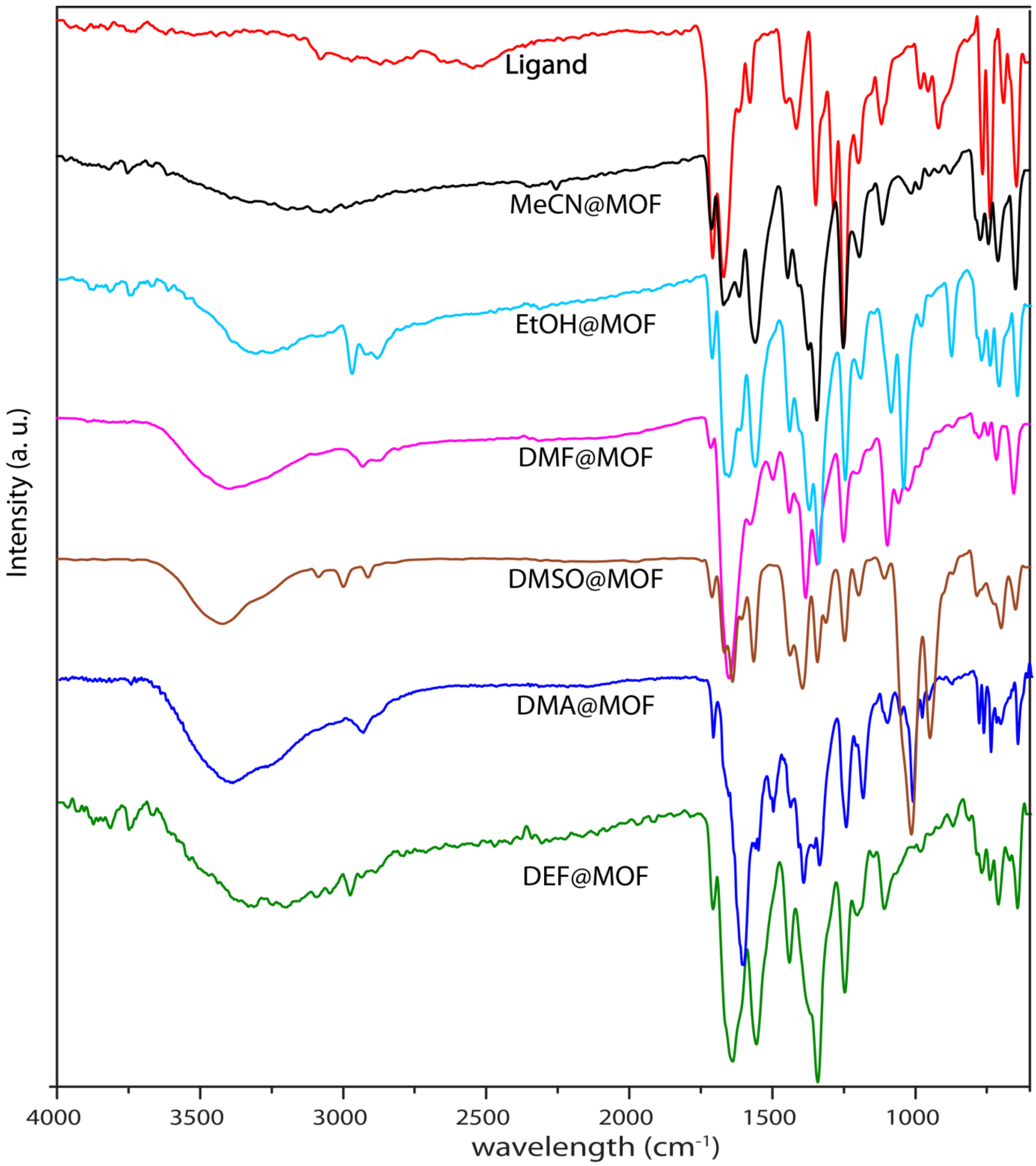

Figure S9: FT-IR spectra of H_4BINDI and solvent incorporated Mg-NDI.

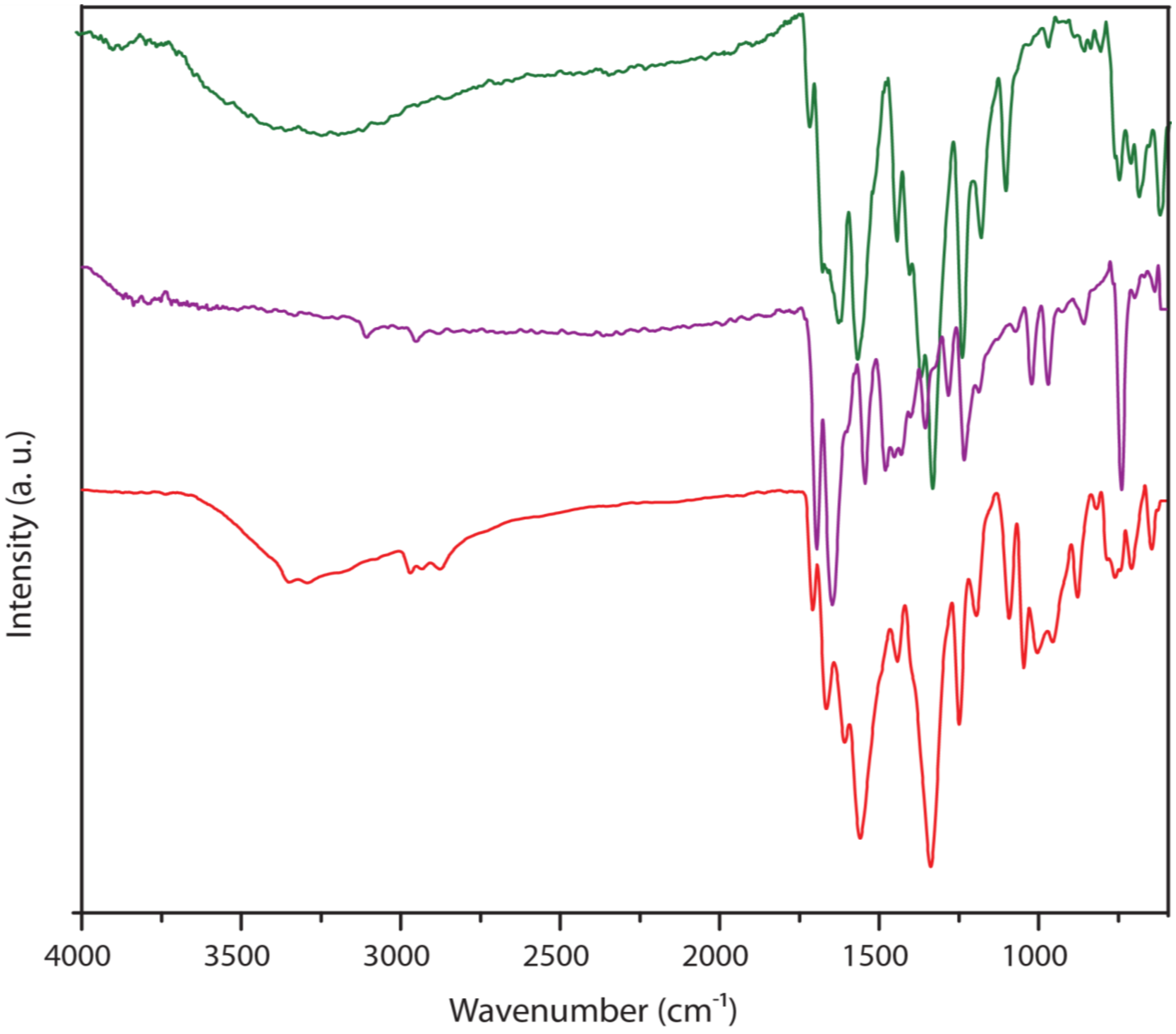

Figure S10: FT-IR spectra of Mg-NDI in presence of hydrazine (red), aniline (purple) and triethylamine (green).

S6: TG analyses of the materials

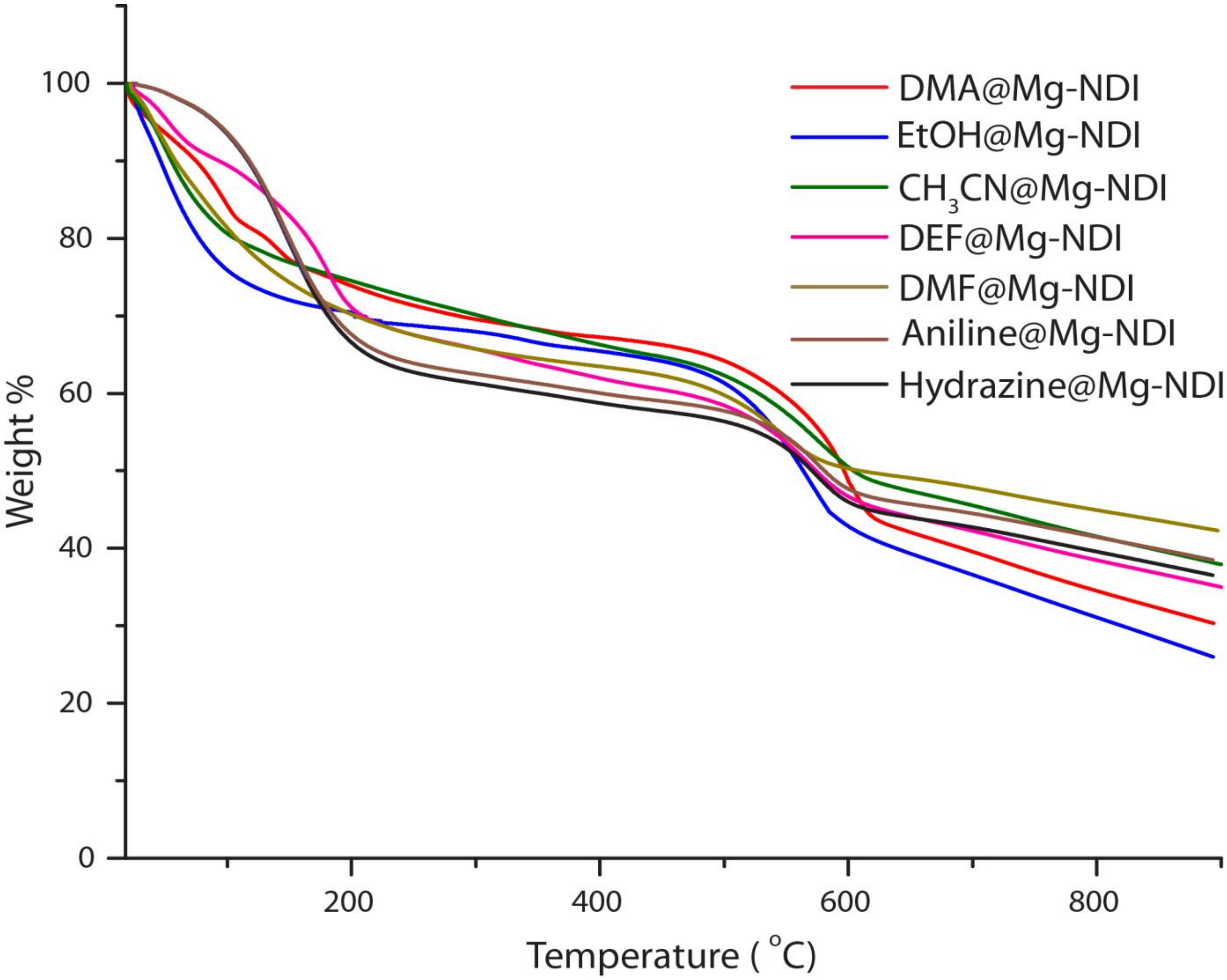

Figure S11: TGA plots of Mg-NDI and different analyte incorporated MOFs.

S7: UV-Vis spectroscopic measurements

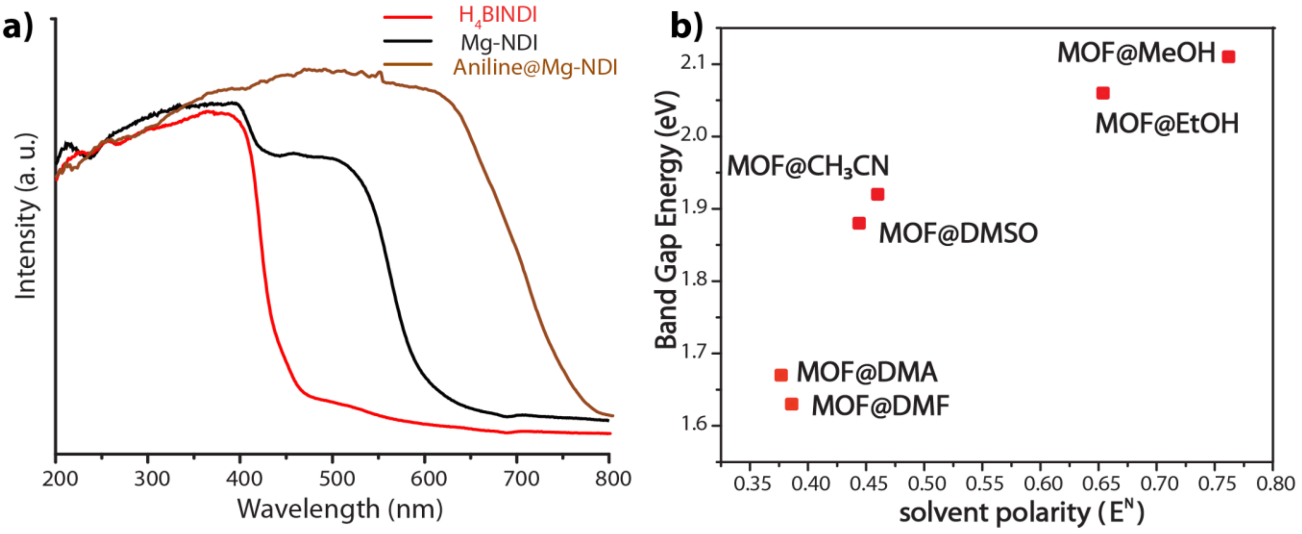

Figure S12: a) Solid state UV-vis spectra of H_4BINDI (red), Mg-NDI (black) and aniline@Mg-NDI (brown). b) Band gap value *vs.* polarity has been plotted, which shows the band gap value increases with increasing polarity.

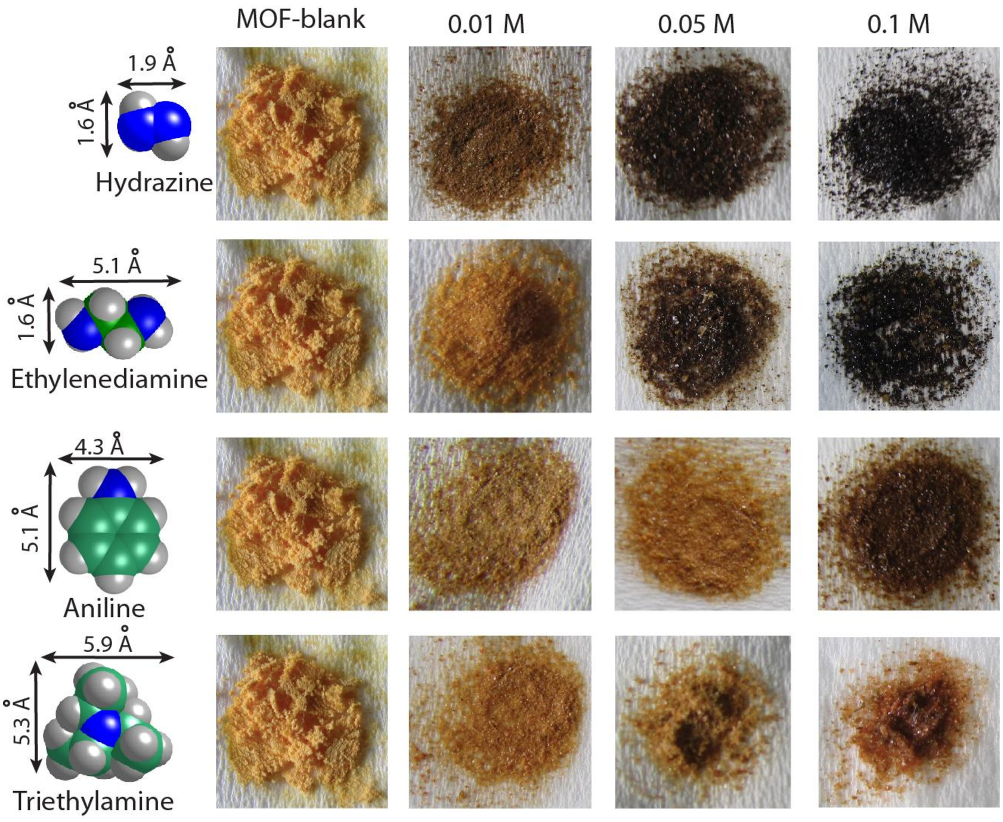

Figure S13: Optical images representing the color change of Mg-NDI in presence of different amines at different concentrations.

S8: Confocal microscopy imaging

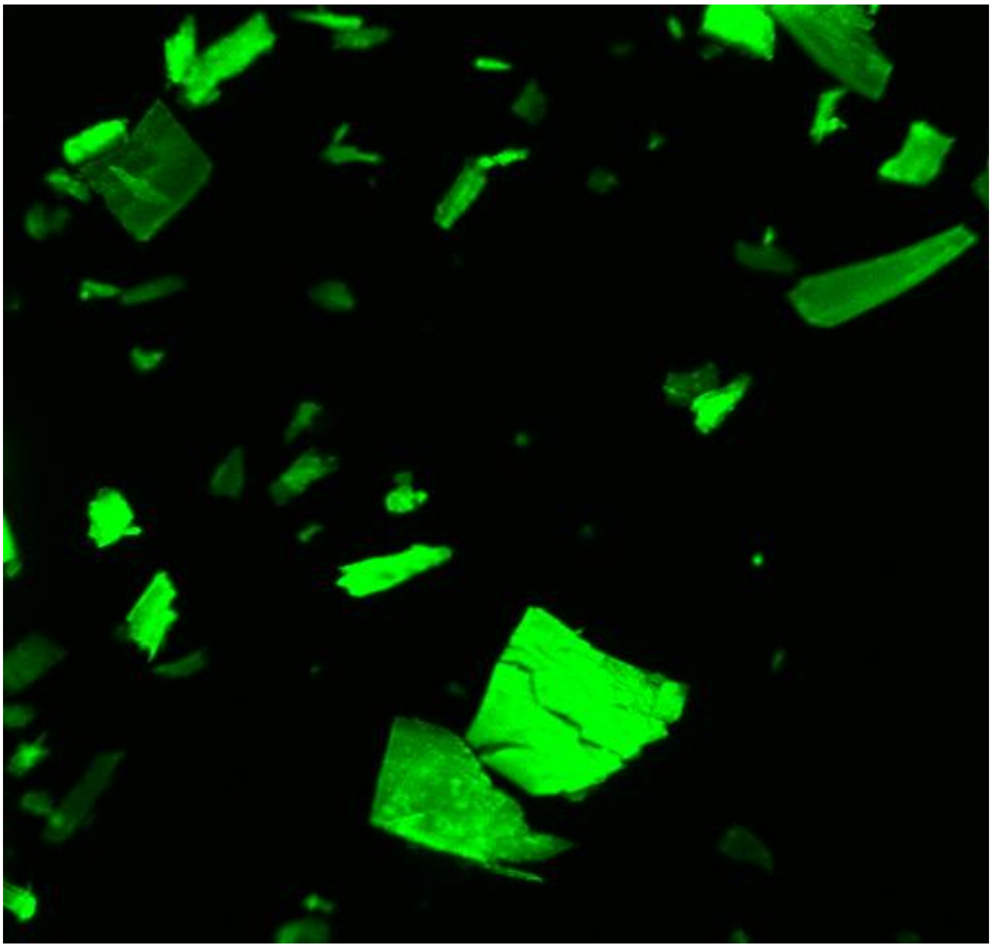

Figure S14: Confocal image of **Mg-NDI** after irradiation with ~515 nm wavelength light source.

S9: Photoluminescence (PL) analysis

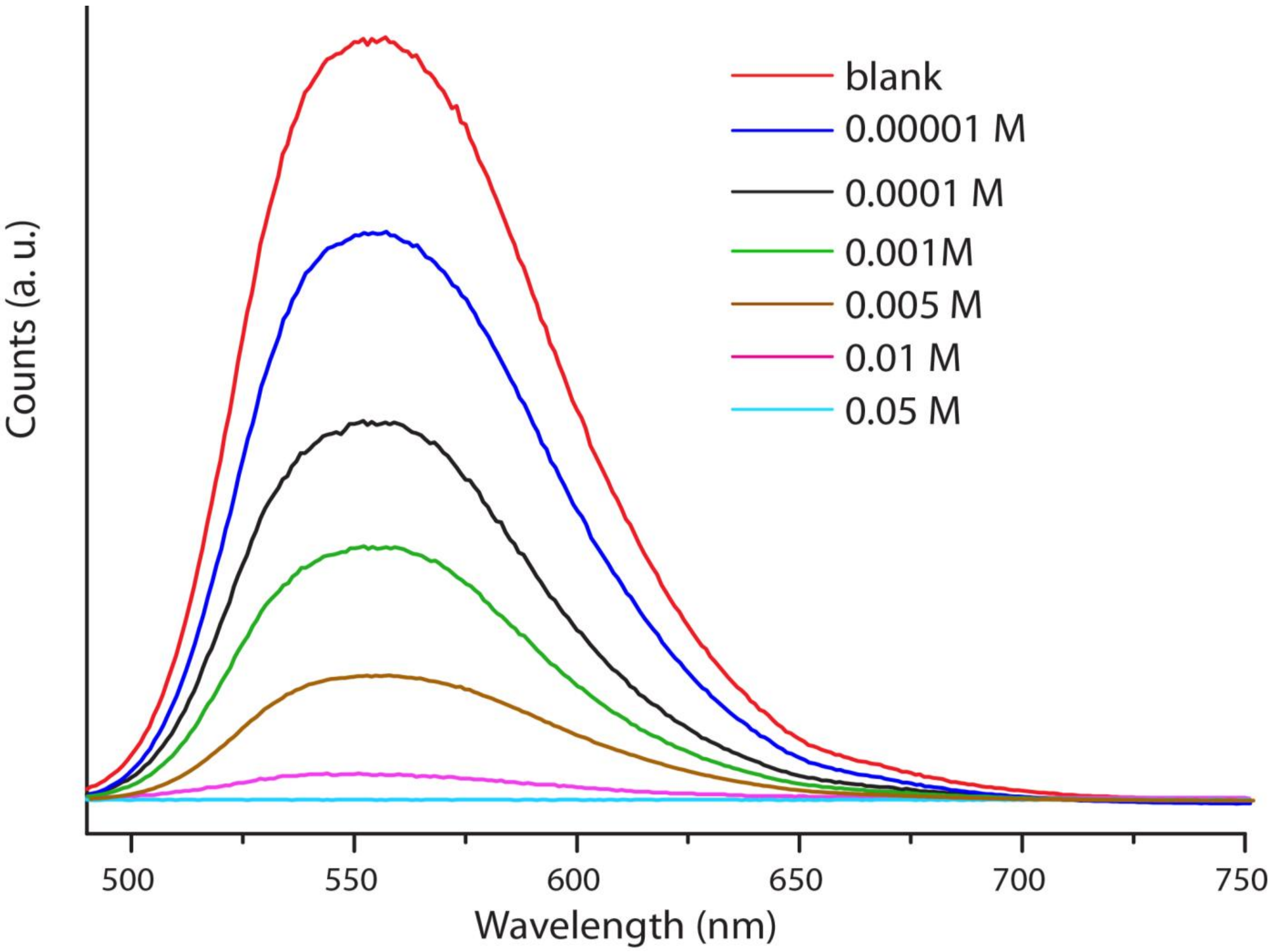

Figure S15: PL quenching spectra of **Mg-NDI** in presence of different concentration of **hydrazine**.

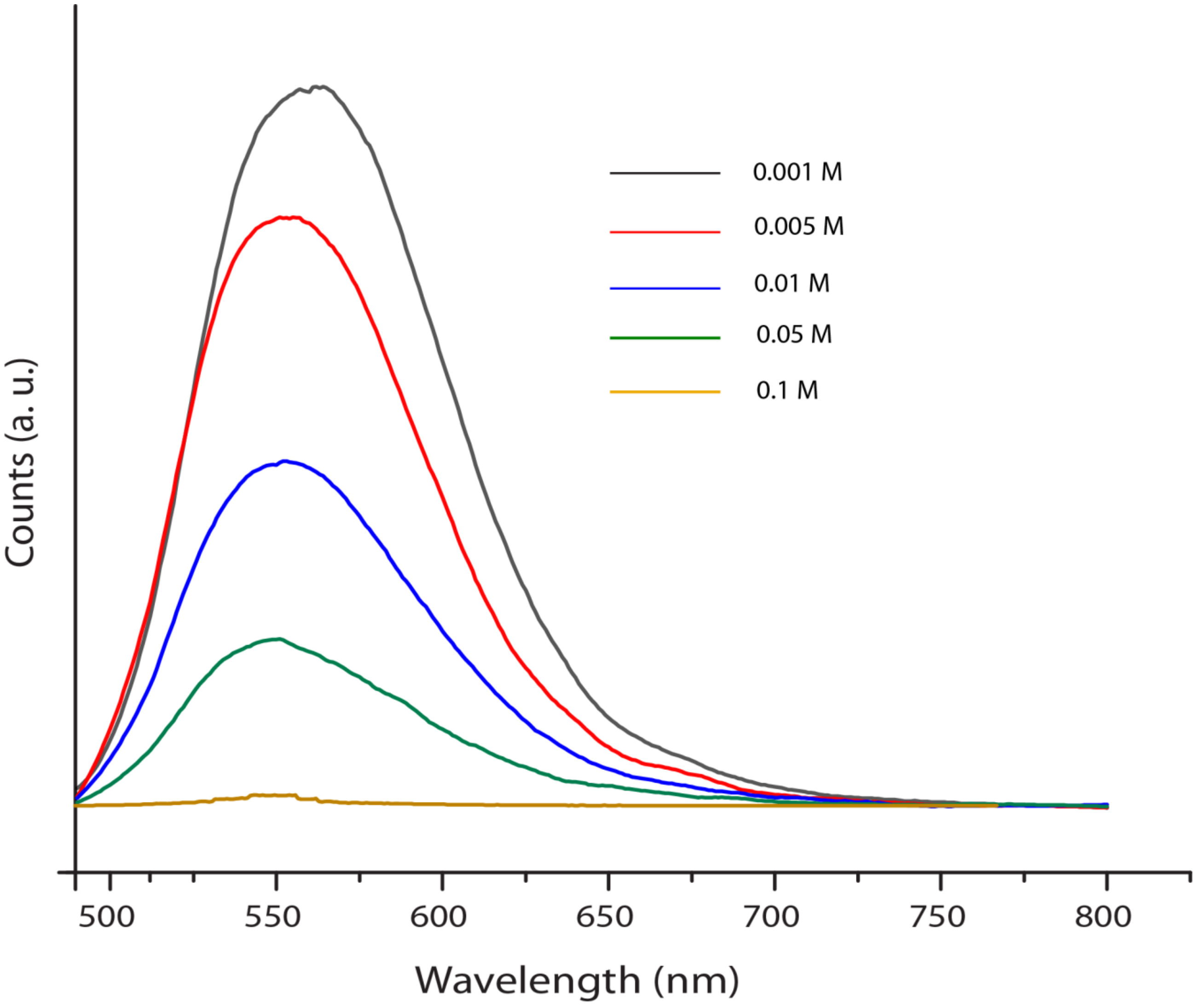

Figure S16: PL quenching spectra of **Mg-NDI** in presence of different concentration of **ethylene-diamine**.

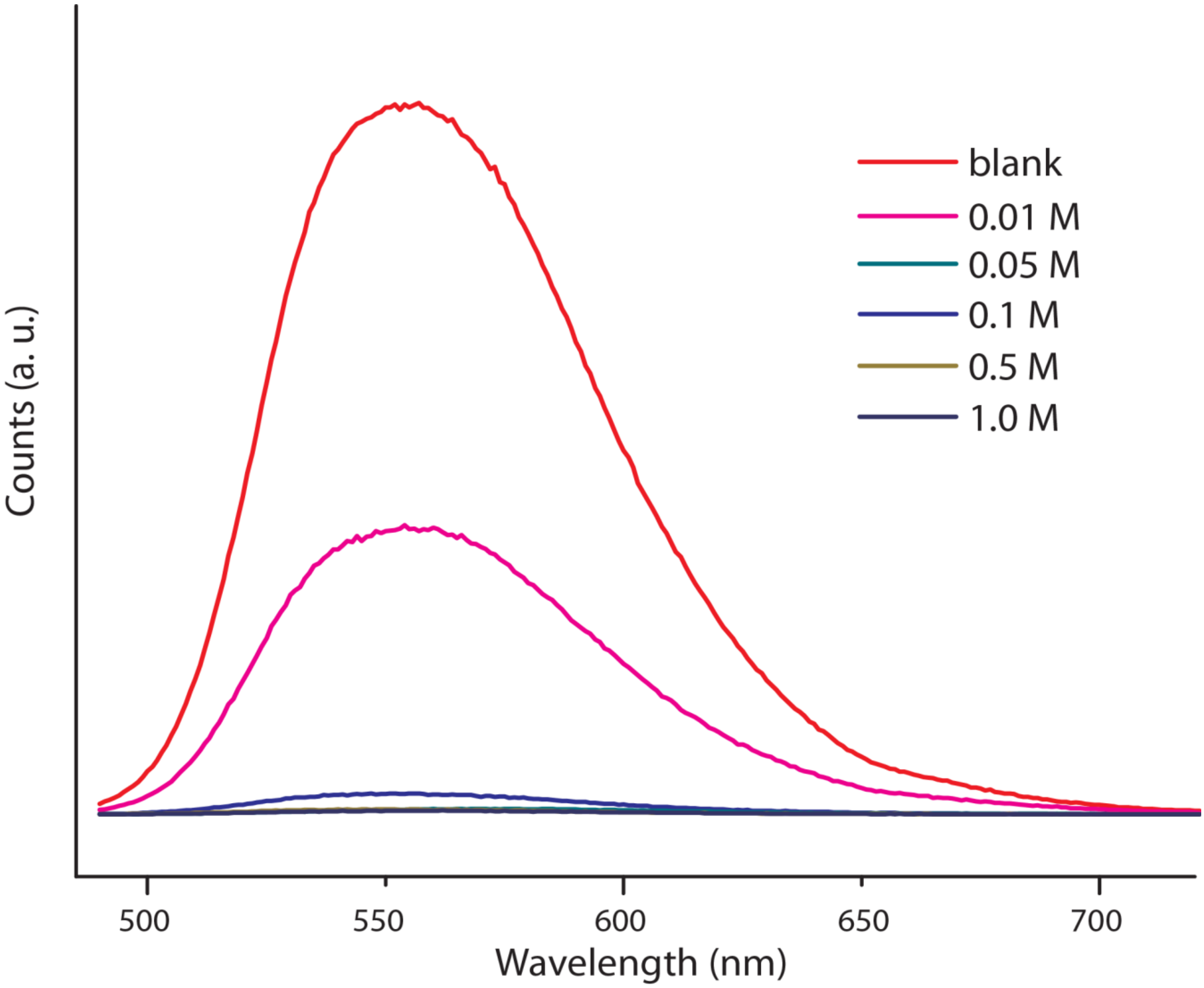

Figure S17: PL quenching spectra of **Mg-NDI** in presence of different concentration of **triethylamine**.

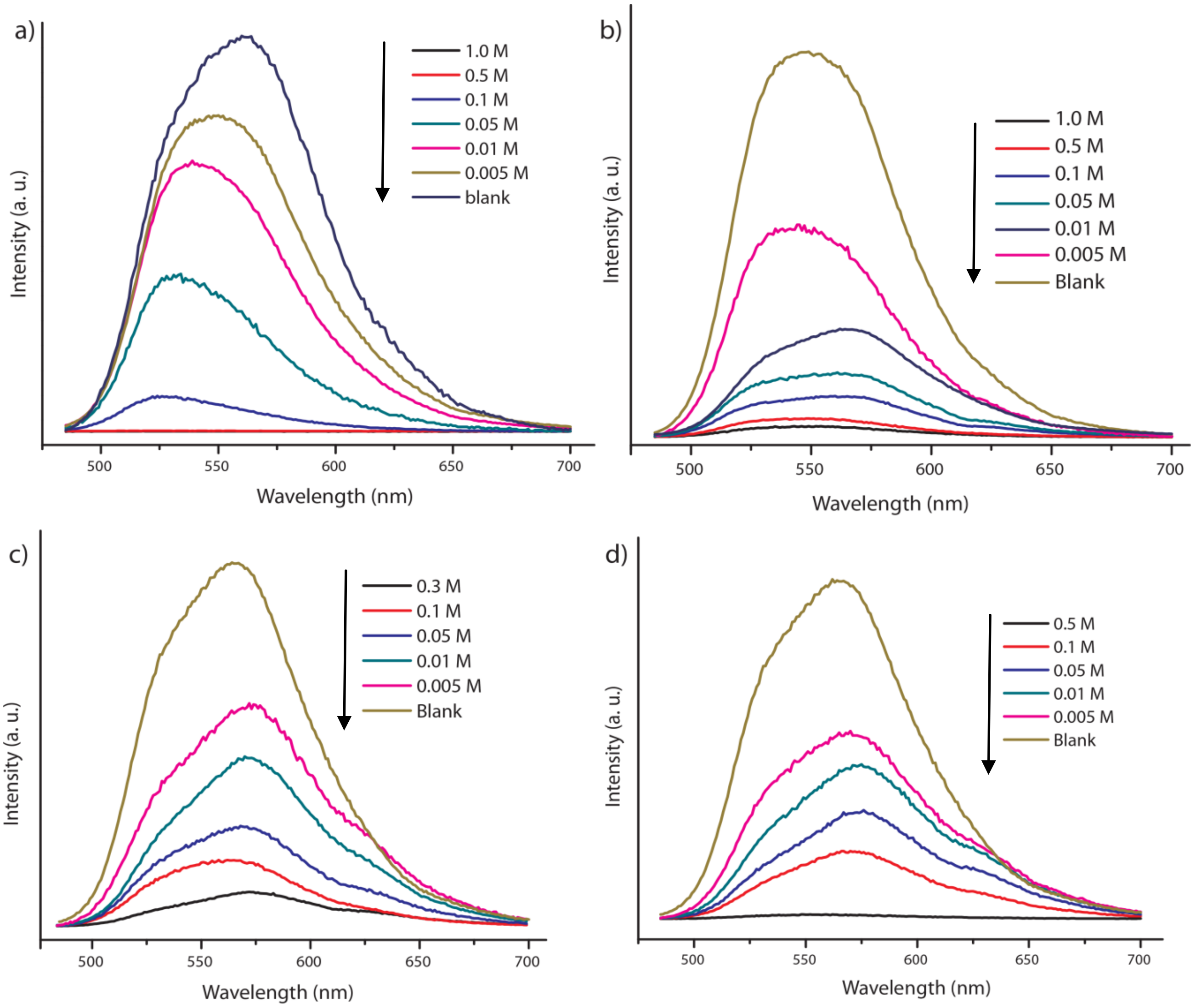

Figure S18: PL quenching spectra of **Mg-NDI** in presence of different concentration of a) **Diethylamine,** b) **1,3-propanediamine,** c) **Ethylamine,** d) **Methylamine**.

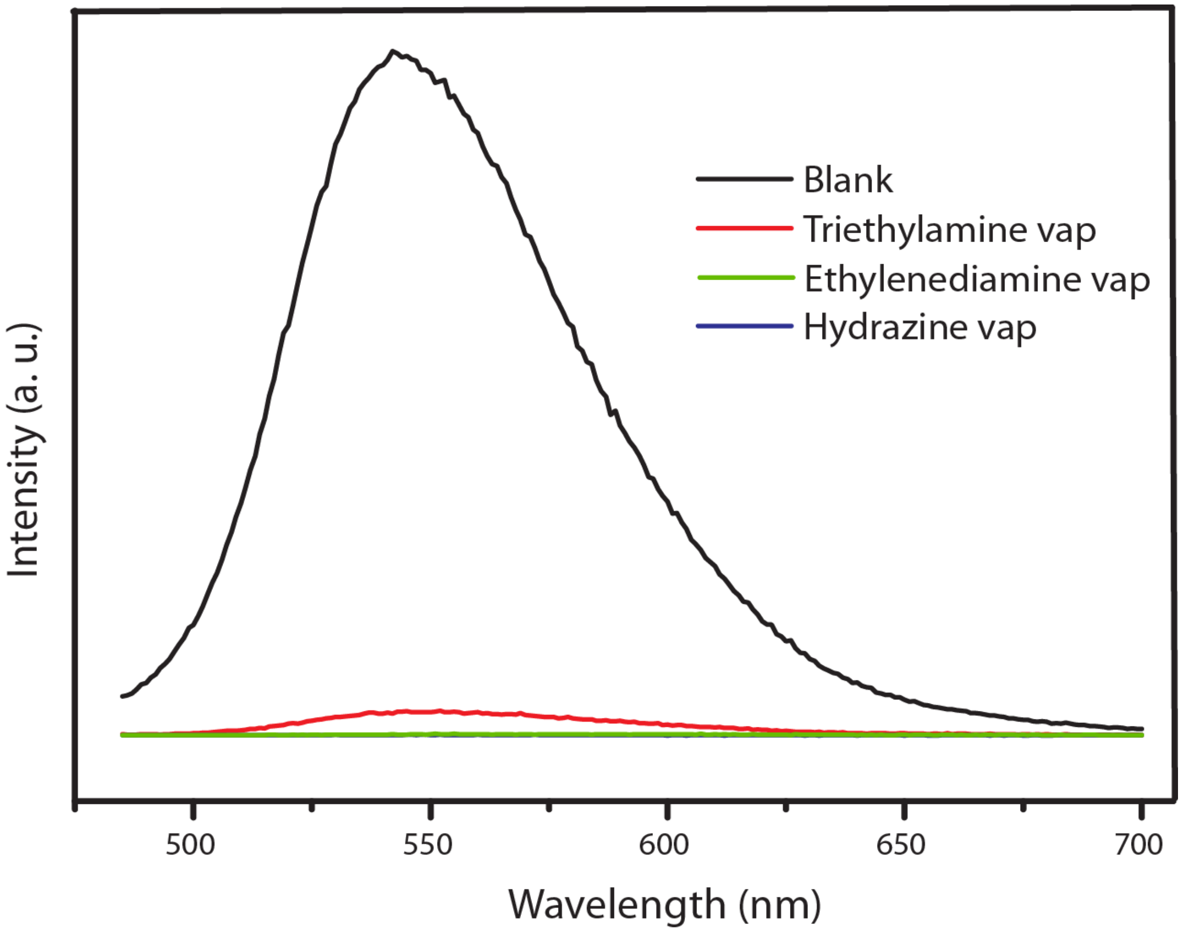

Figure S19: PL quenching spectra of **Mg-NDI** in presence of different amine vapors.

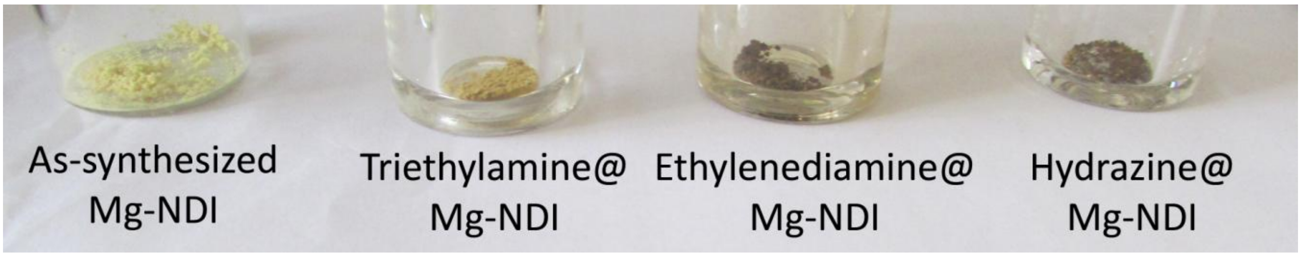

Figure S20: Photograph representing the color change of **Mg-NDI** in presence of different amine vapors.

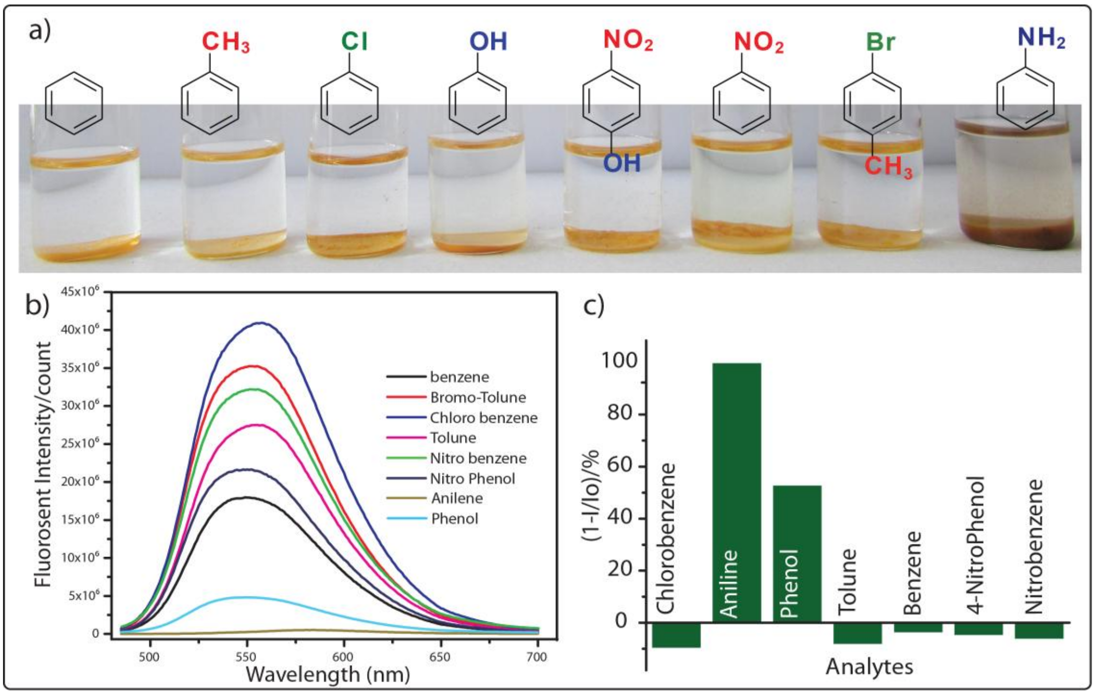

Figure S21: a) Photograph representing the selective color change in case of aniline with respect to other aromatic functional molecules, b) solid state PL spectra of **Mg-NDI** in different aromatic functional molecules, c) bar chart representation for quenching efficiency of **Mg-NDI** in presence of different analytes (0.01 M in EtOH).

S10: N_2 adsorption measurements

In order to understand the interaction of the analyte molecules with the pore walls for showing the solvatochromic property the N_2 adsorption isotherms have been checked of the solvent molecule incorporated Mg-NDI MOF materials. Thus, if there are any solvent molecules trapped inside the MOF pores, the change in N_2 adsorption of the materials can be observed. For this, the as-synthesized Mg-NDI crystals were soaked in the respective solvent(s) for a prolonged time [2 h] and then dried the material under vacuum for 6 h to remove the loosely bound solvent molecules on the crystal surface and in between the crystallites. N_2 adsorption of these dried materials revealed their non-porous nature and the BET surface area was drastically decreased because of the presence of solvent molecules. The following N_2 adsorption isotherms indicate that the respective materials do not contain any pore surfaces accessible for the incoming N_2 molecules. Thus, it can be concluded that this decrease in surface area for the solvent loaded materials compared to the completely evacuated materials is because of the trapped solvent molecules present inside the MOF pores. It is noteworthy that the MOF crystals were vacuum dried with a degasser prior to this study, thus chances of pore blockage by the solvent molecules adsorbed on surface of in the inter-crystallite spaces has been diminished. Therefore, this non porous nature of the solvent incorporated materials concluded that the solvatochromic behavior of Mg-NDI is because of the solvent molecules present and interacting inside the pores of the framework.

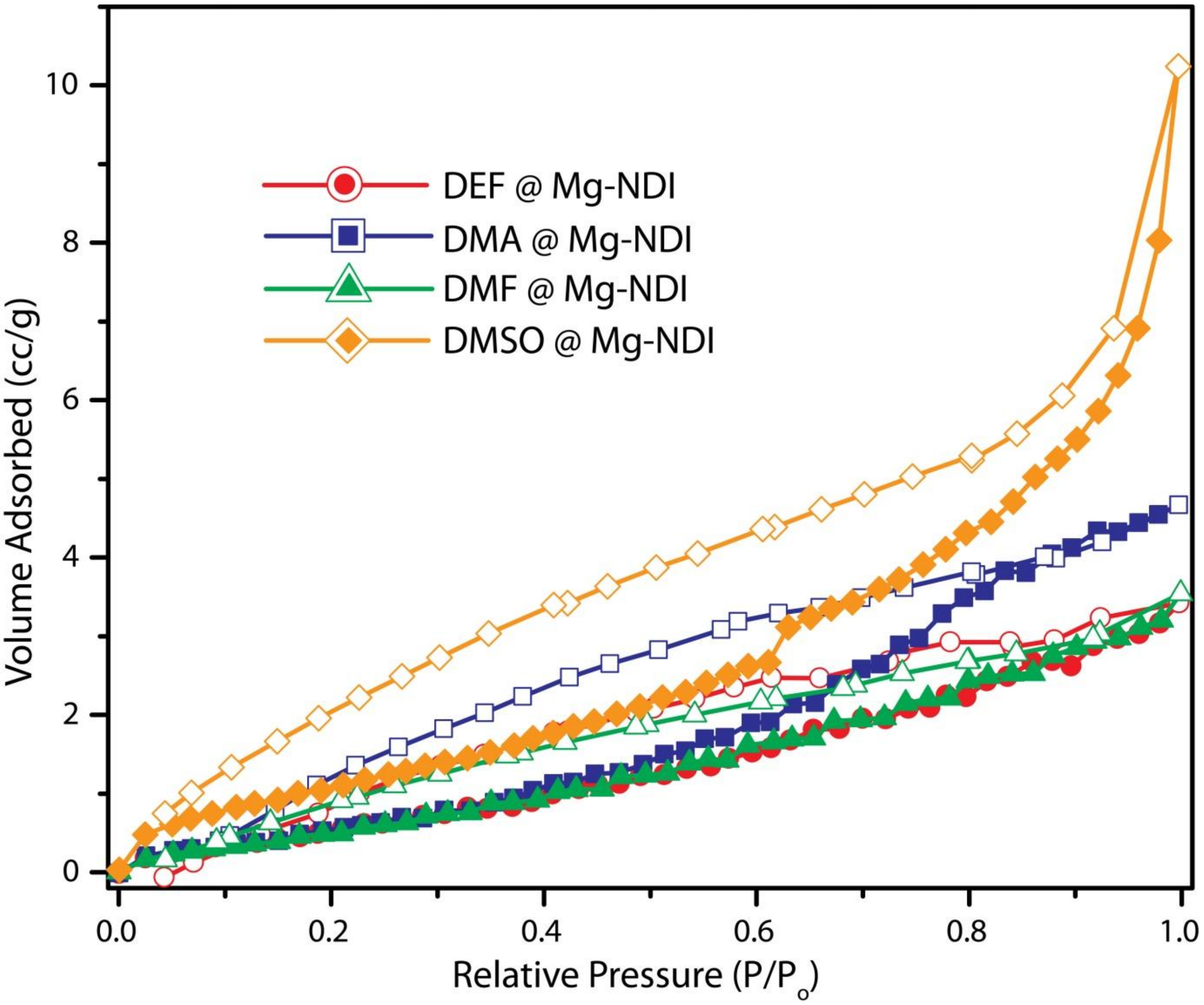

Figure S22: N_2 adsorption isotherms for solvent loaded Mg-NDI at 77 K. The filled and unfilled shapes are representing the adsorption and desorption respectively.

S11: Electrochemical measurements

The reduction potentials of the Mg-NDI were measured using a three-electrode cell at room temperature. A platinum electrode was used as the working electrode, platinum wire as the counter-electrode, and a platinum wire as the reference. Electrochemical measurements of the analytes were carried out using 0.01 mmol solutions of tetrabutylammonium hexafluoro phosphate solution in acetonitrile. For the MOFs, the powdered materials were coated on 3 mm wide platinum electrode. The reduction potentials of the compounds were obtained from the cyclic voltammograms and corrected with respect to the Fc/Fc^+ internal standard.

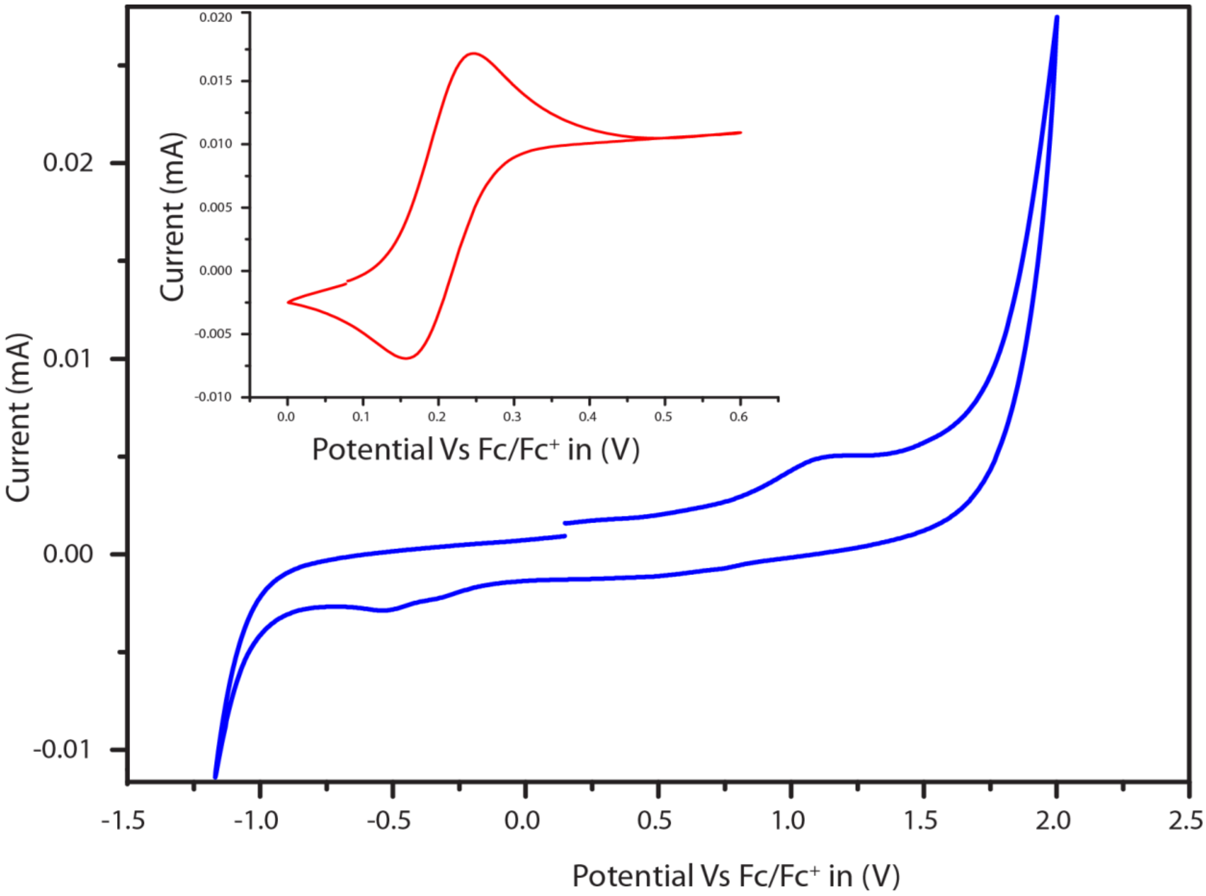

Figure S23: The cyclic voltammograms of **Mg-NDI** in acetonitraile medium and (inset) cyclic voltammogram of ferrocene.

S12: DFT calculation for Mg-NDI

For each solvent@MOF combination, 100 initial geometries were generated using the Kick[3] stochastic structure generator[5]. Positions of the solvent molecules were optimised using DFTB,[6,7] keeping the framework fixed at the experimental geometry. The optimised structures were ranked in terms of energy and the energy levels of the HOMO and LUMO were extracted for the lowest energy structure of each solvent@MOF system.

To generate cube files for the frontier orbitals, single point calculations of the DFTB optimized geometry in periodic boundary conditions were performed at DFT level. Density functional theory (DFT) was applied within the generalized gradient approximation, using the PBE XC functional.[8] The Gaussian and Plane-Wave method, as it is implemented in CP2K package,[9,10] with DZVP-MOLOPT-GTH basis set and Goedecker-Teter-Hutter pseudopotentials[11,12] was applied for all atoms. The charge density cutoff of the finest grid level is equal to 400 Ry. The number of used multigrids is 5.

S13: References

1. (a) Cairns, A. J.; Perman, J. A.; Wojtas, L.; Kravtsov, V. C.; Alkordi, M. H.; Eddaoudi, M.; Zaworotko, M. J. *J. Am. Chem. Soc.* **2008**,*130*, 1560. (b) Nelson, A. P.; Farha, O.K.; Mulfort, K. L.; Hupp, J. T. *J. Am. Chem. Soc.* **2009**, *131*, 458. (c) Perman, J. A.; Cairns, A. J.; Wojtas, L.; Eddaoudi M.; Zaworotko, M. J. *CrystEngComm* **2011**, *13*, 3130. (d) Han, L.; Qin, L.; Xu, L.; Zhou, Y.; Sun, J.; Zou, X. *Chem. Commun.* **2013**, *49*, 406.
2. CrysAlisPro, Version 1.171.33.66; Oxford Diffraction Ltd.: Abingdon, U.K., **2010**.
3. G. M. Sheldrick, (**1997**). *SHELXS '97* and *SHELXL '97*. University of Göttingen, Germany.
4. A. L. Spek (**2005**) PLATON, *A Multipurpose Crystallographic Tool*, Utrecht University, Utrecht, The Netherlands.
5. Addicoat, M. A.; Fukuoka, S.; Page, A. J.; Irle, S.; *J Comput Chem.***2013**, *30*, 2591.
6. Aradi, B.; Hourahine, B.; Frauenheim, T. DFTB+, a sparse matrix-based implementation of the DFTB method. *J. Phys. Chem.* **2007**, A 111, 5678.
7. Wahiduzzaman, M.; Oliveira, A. F.; Philipsen, P.; Zhechkov, L.; van Lenthe, E.; Witek, H. A.; Heine, T.; *J. Chem. Theory Comput.* **2013**, *9*, 4006.
8. Perdew, J. P.; Burke, K.; Ernzerhof, M. *Phys. Rev. Lett.* **1996**, *77*, 3865.
9. Vondele, J. V.; Krack, M.; Mohamed, F.; Parrinello, M.; Chassaing, T.; Hutter, J. *Comput. Phys. Commun.* **2005**, *167*, 103.
10. The CP2K developers group, http://www.cp2k.org/ **2012**.
11. Goedecker, S.; Teter, M.; Hutter, J. (**1996**) Separable dual-space Gaussian pseudopotentials, *Phys. Rev.* B54, 1703.
12. Hartwigsen, C.; Goedecker, S.; Hutter, J. (**1998**) Relativistic separable dual-space Gaussian pseudopotentials from H to Rn, *Phys. Rev.* B58, 3641.
